# Effects of magnesium potassium sulfate on Tibetan sheep quality and its volatile and non-volatile metabolic substances

**DOI:** 10.3389/fnut.2026.1821555

**Published:** 2026-05-11

**Authors:** Yuan Wang, Lijuan Han, Shengzhen Hou, Linsheng Gui, Zhenzhen Yuan, Shengnan Sun, Zhiyou Wang, Baochun Yang, Chao Yang

**Affiliations:** College of Agriculture and Animal Husbandry, Qinghai University, Xining, China

**Keywords:** meat quality, potassium magnesium sulfate, Tibetan sheep, untargeted metabolomics, volatile flavor compounds

## Abstract

**Introduction:**

Preliminary research has identified that potassium and magnesium may influence the quality of Tibetan sheep meat, although the underlying mechanisms remain unclear.

**Methods:**

This study investigated the effects of dietary supplementation with magnesium potassium sulfate (PMS) on carcass quality, meat quality, nutritional composition, and flavor metabolites of Tibetan sheep, and explored potential mechanisms using a metabolomics approach.

**Results:**

Dietary inclusion of 0.3% PMS significantly enhanced carcass quality, as shown by increases in abdominal wall thickness and backfat thickness by 13.13% and 7.41%, respectively, compared with the control group (*p* < 0.05). Supplementation with 0.25% PMS significantly elevated pH at 24 h, a, and intramuscular fat content, while reducing pH at 45 min and b (*p* < 0.05). The 0.25% PMS group also exhibited significantly higher concentrations of linoleic acid, linolenic acid, EPA, and DHA than the control group (*p* < 0.05). Volatile compound analysis showed that PMS was associated with the production of fruity and nutty compounds (e.g., methyl 3-methylpentanoate and methyl heptanoate) and with the suppression of malodorous substances such as 1-hydrazinopropan-2-ol. Non-volatile metabolomics revealed that PMS supplementation was associated with enhanced fatty acid biosynthesis, which correlated with the adipocyte cytokine signaling pathway.

**Discussion:**

PMS supplementation was further associated with modulation of the sphingolipid signaling pathway, which correlated with synergistic regulation of meat quality and contributed to an improved flavor profile of Tibetan sheep meat.

## Graphical abstract

**Figure:**
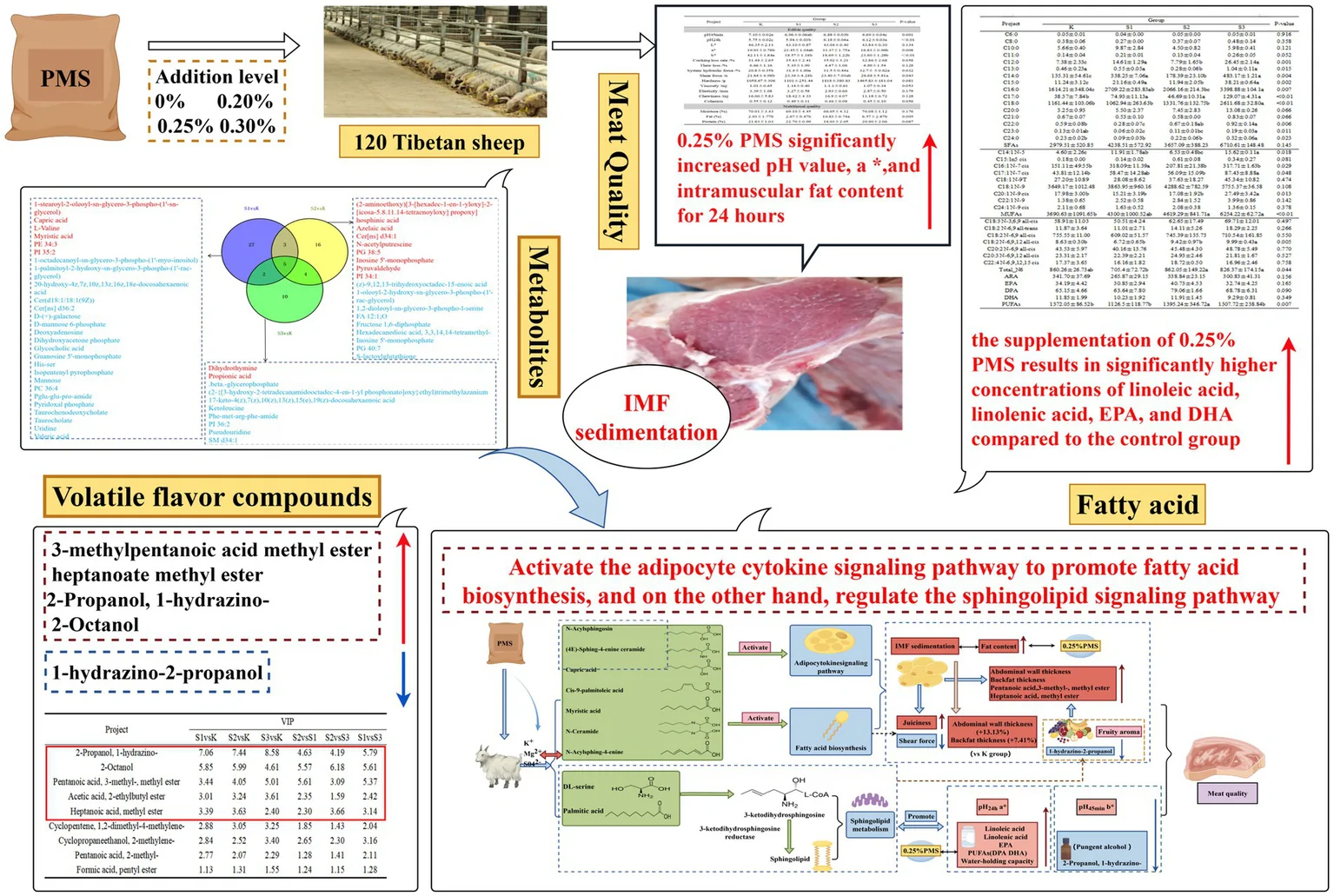
Summary of the effects of adding PMS on Tibetan sheep quality, volatile metabolites, and non-volatile metabolites. The blue arrow indicates a significant decrease in substance content (*p* < 0.05), while the red arrow indicates a significant increase in substance content (*p* < 0.05). Group K was fed with feed without PMS, Group S1 was fed with feed containing 0.20% PMS, Group S2 was fed with feed containing 0.25% PMS, and Group S3 was fed with feed containing 0.30% PMS.

## 1 Introduction

Tibetan sheep, also referred to as Tibetan-type sheep, represent one of China’s three principal primitive sheep breeds. These sheep are predominantly found across the Qinghai–Tibet Plateau and adjacent frigid regions at elevations exceeding 3,000 meters. As a result, they have developed traits such as resistance to extreme cold, the ability to subsist on coarse feed, a robust constitution, disease resistance, proficiency in navigating steep terrains and covering long distances, as well as strong foraging abilities. Based on their ecological environment and production/economic characteristics, Tibetan sheep are primarily categorized into three types: plateau (grassland), valley, and Oula. Their meat is characterized by its tenderness and rich aroma, and it is noted for its high protein, low fat, and low cholesterol content (1). Traditionally, Tibetan sheep were predominantly raised through grazing practices. However, recent rapid economic development has led to an increased market demand for Tibetan sheep. Simultaneously, overgrazing has resulted in decreased vegetation coverage in alpine meadows and a reduction in soil’s water retention capacity. The grazing and trampling activities of Tibetan sheep exacerbate freeze–thaw erosion, thereby contributing to localized desertification within the fragile ecosystems of Qinghai (2). As a result, large-scale stall feeding is increasingly being adopted within the livestock industry (3).

Unlike traditional grazing, stall feeding permits the artificial regulation of the nutritional intake of Tibetan sheep. This method not only fulfills the growth and developmental requirements of Tibetan sheep but also improves slaughter performance, edible quality, and nutritional value, thereby emerging as a progressive trend in Tibetan sheep farming (1). Common feedstuffs used in stall feeding are generally classified into roughage and concentrate feeds. Roughage is characterized by its low nutritional value, poor palatability, and coarse texture (4). In contrast, concentrate feeds are richer in nutrients per unit volume compared to roughage, particularly in terms of amino acids, fatty acids, and mineral elements, while maintaining a low crude fiber content. The mineral content is a standard measure for assessing the nutritional quality of concentrates and serves as a critical indicator in ruminant-specific compound feeds. Research conducted by da Silva Diniz et al. (5) demonstrates that mineral elements, as essential components of animal feed, play a significant role in influencing both meat quality and metabolic processes in animals. The study suggests that sensory attributes such as taste, flavor, juiciness, and tenderness are correlated with the content of minerals such as iron (Fe), magnesium (Mg), manganese (Mn), phosphorus (P), and selenium (Se) (6). In Angus cattle, there are moderate to strong correlations between potassium (K) and iron (Fe) levels and the presence of cholesterol, monounsaturated fatty acids (MUFA), and polyunsaturated fatty acids (PUFA) (7). Similarly, piglets supplemented with zinc exhibit increased intramuscular fat (IMF) deposition, attributed to the upregulation of genes involved in lipid synthesis and fatty acid transport (8).

Traditional dietary beliefs in Qinghai Province suggest that Tibetan sheep raised in saline-alkali environments produce meat of superior quality compared to those from non-saline-alkali areas, characterized by a tender texture and milder gaminess (9). However, this belief currently lacks substantial empirical support. Qinghai Province is characterized by its vast expanse and the extensive distribution of saline-alkali land. Preliminary investigations conducted by the research team indicate that a comparative analysis of the meat texture and flavor of Tibetan sheep reared on saline-alkali land versus non-saline-alkali land reveals that minerals such as potassium, magnesium, and sodium, along with elevated concentrations of sulfate ions prevalent in the grass, soil, and water of saline-alkali regions, may be critical factors in enhancing the quality of Tibetan sheep meat (10, 11). Potassium, the primary cation in intracellular fluid, is the most significant macromineral after calcium and phosphorus within animals, playing an essential role in maintaining cellular osmotic pressure, acid–base balance, and neuromuscular excitability. Magnesium, identified as the seventh most abundant mineral element in the body (12), is involved in over 300 enzymatic reactions and is crucial for physiological processes such as energy metabolism, protein synthesis, and nerve conduction. In conjunction with potassium and sodium, magnesium is vital for maintaining muscle and nerve excitability, sustaining acid–base and electrolyte balance, and mitigating animal stress. Sulfur, while an essential mineral for life, cannot be stored in animal bodies and must be supplied through dietary intake. In livestock and poultry diets, sulfates and sulfur-containing amino acids serve as the primary sources of sulfur. Research indicates that incorporating 2.5% mica magnesium into the diet of fattening pigs can enhance meat yield (13). Concurrently, magnesium supplementation has been shown to reduce drip loss and improve meat color. As a meat quality enhancer, magnesium also contributes to extending muscle shelf life to some extent (9). Meat treated with MgSO₄ demonstrates reduced cooking losses and improved sensory attributes, such as juiciness, tenderness, and overall acceptability (14). Although potassium and magnesium can independently enhance meat quality, they typically require high dosages. However, their combined use allows for reduced individual dosages (15).

PMS, a novel mineral feed supplement comprising magnesium, potassium, and sulfur compounds, has been developed (16). Dietary supplementation. On February 20, 2024, it was reported that following an evaluation by the National Feed Review Committee, organized by the Ministry of Agriculture and Rural Affairs, PMS, developed by Qinghai Blue Lake Shencheng Biotechnology Co., Ltd., received official approval as a new feed additive (Ministry of Agriculture and Rural Affairs Announcement No. 744). As a composite mineral additive containing magnesium and potassium, PMS presents distinct potential applications in animal nutrition (17). Research suggests that the inclusion of 0.30% PMS in the diet of weaned piglets, as demonstrated by Cao et al. (16), enhances feed intake, boosts intestinal antioxidant capacity, and improves gut microbiota composition. Panella-Riera et al. (18) reported that the supplementation of pig diets with magnesium sulfate significantly decelerated pH decline at 45 min post-mortem and reduced drip loss. Furthermore, magnesium and potassium have been shown to exert synergistic effects in enhancing animal antioxidant defenses and mitigating disease-related damage. Obese rats on a high-fat diet supplemented with seawater rich in potassium, magnesium, and calcium exhibited reduced hepatic triglyceride and cholesterol levels (19). While current research has explored the application of PMS in the diets of pigs and other species, investigations into the effects of PMS on the meat quality of Tibetan sheep have yet to be reported.

In order to elucidate the mechanisms underlying the variations in meat quality of Tibetan sheep attributable to different levels of PMS supplementation in their diets, this study utilized Tibetan sheep as experimental subjects. A feeding trial was structured into four distinct groups based on PMS supplementation levels, with samples of the longissimus dorsi muscle collected for comprehensive analysis of edible quality, nutritional attributes, mineral content, and both targeted and untargeted metabolites. The study aimed to elucidate the relationship between PMS-induced differential metabolites and the quality of Tibetan sheep meat, identify key metabolites and pathways through which PMS exerts its influence on meat quality, and systematically assess the effects of PMS-a natural mineral supplement abundant in potassium and magnesium-on the meat quality of Tibetan sheep. Additionally, this research provides a theoretical foundation for the regulation of meat quality in high-altitude pastoral regions.

## 2 Materials and methods

The Committee of Experimental Animal Care approved all experimental procedures involving animals, while the Qinghai University of Animal Care approved handling techniques (QUA-2022-0515).

### 2.1 Animal husbandry

In this experiment, 120 healthy, similarly conditioned, 2-month-old weaned female Tibetan sheep were selected. The lambs were stratified by initial body weight (mean ± standard deviation), and within each stratum, they were randomly assigned to four treatment groups (K, S1, S2, S3), with 30 lambs per group. Allocation concealment was achieved using sealed envelopes prepared by a researcher who was not involved in subsequent experiments. Group K functioned as the control group and was provided with a basal diet that included a concentrate supplement, silage oat hay, and green-dried oat hay. The concentrate supplement was primarily composed of corn, soybean meal, and a premix. The experimental groups, designated as S1, S2, and S3, received the basal diet with the addition of PMS at concentrations of 0.2, 0.25, and 0.3%, respectively, to the concentrate supplement. Table 1 provides a detailed account of the composition and nutritional levels of the concentrate supplement in each group’s diet. The formal feeding trial lasted for 120 days, preceded by a 7-day adaptation period. All lambs were 2 months old at the beginning of the trial and were slaughtered at approximately 6 months of age. All lambs were housed in a fully enclosed barn with distinct pens, which included an exercise yard and offered sheltered, sunny, dry, and well-ventilated conditions. Feeding occurred twice daily at 8:30 a.m. and 4:30 p.m., with lambs having ad libitum access to feed and water.

**Table 1:** Dietary composition and nutritional level (dry matter basis %).

Dietary composition	K (%)	S1 (%)	S2 (%)	S3 (%)
Corn	51	50.8	50.75	50.7
Wheat	10	10	10	10
Soybean meal	4	4	4	4
Rapeseed meal	14	14	14	14
Palm meal	17	17	17	17
Salt	1	1	1	1
Stone powder	1	1	1	1
Baking soda	1	1	1	1
1% premix (lamb)	0.6	0.6	0.6	0.6
4% concentrate (No. 3)	0.4	0.4	0.4	0.4
Magnesium potassiumsulfate	0	0.2	0.25	0.3
Total	100	100	100	100

Upon completion of the feeding trial, six lambs from each group were randomly selected and transported to Qinghai Xiangsanjang Animal Husbandry Co., Ltd. for slaughter. In accordance with animal welfare protocols, the animals were subjected to a 24-h fasting period and a 2-h water deprivation period prior to slaughter. All animals were slaughtered according to Halal procedures without prior anesthesia, in compliance with local animal welfare regulations and religious practices. The longissimus dorsi muscle, located between the 12th and 13th ribs, was excised, meticulously trimmed of fat and fascia, placed on dry ice, transported to the laboratory, and stored at −80 °C for subsequent analysis.

PMS supplement: Potassium magnesium sulfate was obtained from Qinghai Lanhu Shancheng Biotechnology Co., Ltd. (Batch No. 20260328). According to the certificate of analysis, the product contained 11.17% Mg, 22.79% K, and 22.14% S, with a moisture content of 0.31%. The certificate of analysis is provided as Supplementary Figure S4.

### 2.2 Meat quality analysis

#### 2.2.1 Carcass quality assessment

The assessment of carcass quality in Tibetan sheep encompassed measurements of withers height, body length, chest circumference, live weight, carcass weight, dressing percentage, eye muscle area, abdominal wall thickness, and backfat thickness, following the methodology of Ma et al., with slight modifications (10).

#### 2.2.2 Food quality determination

Food quality evaluation was conducted using a modified approach based on Xin et al. (11). The pH meter (PHS-3C, Shanghai Laiqi Instrument Factory, Shanghai, China) was calibrated using standard buffer solutions (pH 4.00 and 7.00) at 20 ± 1 °C before each measurement session, following the manufacturer’s instructions. The muscle samples were allowed to equilibrate to room temperature (20 ± 1 °C) prior to pH measurement, and the muscle temperature was confirmed using a thermometer. All measurements were performed in triplicate, and the average values were reported. Color parameters (L^*^, a^*^, b^*^) were measured using an automatic colorimeter (ADCI-60-C, Beijing Chentai Ke Instrument Technology Co., Ltd., Beijing, China). The instrument was set to the D65 illuminant and a 10° observer angle according to CIE standards. The colorimeter was calibrated using a standard white plate before each measurement session. All measurements were performed in triplicate on the surface of the longissimus dorsi muscle after a 30-min blooming period, and the average values were reported. Tenderness was quantified utilizing a tenderness analyzer (Model RH-N50, Guangzhou Runhu Instrument Co., Ltd., Guangzhou, Guangdong, China), whereas water-holding capacity was evaluated with a water-holding tester (Model RH-1000, Guangzhou Runhu Instrument Co., Ltd., Guangzhou, Guangdong, China). The parameters of hardness, elasticity, adhesion, cohesiveness, and chewiness were determined using a texture profile analysis (TPA) texture analyzer (Model TA.XTC-18, Shanghai Baosheng Industrial Development Co., Ltd., Shanghai, China). The analyzer was equipped with a cylindrical probe (P/50, 50 mm diameter). The TPA parameters were set as follows: compression rate of 1.0 mm/s, strain (deformation) of 50%, and 2 cycles with a 5-s interval between cycles. The trigger force was set to 5 g. Samples were compressed twice to 50% of their original height. All measurements were performed in triplicate, and the parameters were calculated from the force-time curves.

#### 2.2.3 Determination of nutritional quality

The moisture, crude fat, and crude protein content of meat samples were determined using the Pettinati method (20).

#### 2.2.4 Determination of mineral element content

The concentration of mineral elements in meat samples was measured by inductively coupled plasma optical emission spectroscopy (ICP-OES, Optima 8300, Perkin Elmer, Waltham, MA, United States) (11).

#### 2.2.5 Sensory evaluation

Sensory evaluation was conducted according to the method of Vivek et al. (21) using a blinded procedure. Twelve panelists (6 males and 6 females, aged 22–30 years) were recruited from food science graduate students who had received standardized training. All panelists underwent three rounds of training to familiarize themselves with the scoring criteria for each sensory attribute (appearance, texture, flavor, juiciness, and overall acceptability). Meat samples were cut into 2 cm × 2 cm × 2 cm cubes, randomly assigned three-digit codes, and presented in random order under blind conditions, with panelists unaware of the treatment group assignments. The evaluation was performed under white light at room temperature (22 ± 2 °C). Each sample was evaluated three times, and the average value was used for analysis. Panelists rinsed their mouths with purified water between samples to eliminate residual flavors. Scoring for appearance, texture, flavor, juiciness, and overall evaluation of Tibetan sheep meat was performed using the criteria provided in Supplementary Table S1. Informed consent was obtained from all participants prior to the sensory evaluation, and the consent form is provided as Supplementary material. Data analysis was performed by a researcher who remained blinded to the group identities throughout the analysis.

### 2.3 Amino acid content determination

Weigh 50 mg into a centrifuge tube. Add a methanol solution (purity ≥99.0%, Fisher Chemical, Pittsburgh, PA, United States), acetonitrile (purity ≥99.0%, Fisher Chemical, Pittsburgh, PA, United States), and an aqueous solution in a 2:2:1 ratio, totaling 0.5 mL. Vortex the mixture using a QT-1 vortex mixer (Shanghai Kit Analytical Instrument Co., Ltd., Shanghai, China) and sonicate it with a JP-100 sonicator (Shenzhen Jiemeng Cleaning Equipment Co., Ltd., Shenzhen, Guangdong, China). Allow the mixture to stand until protein precipitation is observed. Subsequently, vacuum-dry the supernatant using an FD-IC-50 vacuum dryer (Shanghai Bilang Instruments Co., Ltd., Shanghai, China) for future use.

### 2.4 Determination of fatty acid content

Weigh 50 mg of sample and dissolve in a 2:1 dichloromethane (≥99.0%, Sigma, St. Louis, MO, United States)-methanol (≥99.0%, Fisher Chemical, Pittsburgh, PA, United States) solution. Vortex to mix thoroughly. Add 2 mL ultrapure water for washing, then transfer the lower layer of solution and evaporate to dryness under nitrogen. Add 2 mL n-hexane (chromatographic grade, Sinopharm Chemical Reagent Co., Ltd., Shanghai, China), add internal standard, methylate for 0.5 h, add 2 mL ultrapure water, aspirate 1,000 μL supernatant, evaporate to dryness under nitrogen, redissolve in n-hexane, transfer supernatant to injection vial, and analyze by GC-MS (Agilent, Santa Clara, CA, United States).

### 2.5 Determination of volatile metabolic compounds and their concentrations in Tibetan sheep muscle

Accurately weigh 2.5 grams of the sample into a 15 mL headspace vial. Introduce a saturated 20% NaCl solution to facilitate salting out, then seal and label the vial before proceeding with the analysis. The headspace conditions involved placing the sample on a magnetic stirrer (CL19-1, Shanghai Sile Instruments Co., Ltd., Shanghai, China) at 60 °C for 30 min. Subsequently, a pre-conditioned (250 °C for 5 min) 75 μm CAR/PDMS manual extraction fiber was inserted into the headspace vial for a 30-min adsorption period. The extraction fiber was then introduced into the GC-MS injection port for desorption over a duration of 600 s. The samples were analyzed using an Agilent 7890B gas chromatograph coupled with a time-of-flight mass spectrometer, equipped with an Agilent DB-WAX capillary column (30 m × 0.25 mm × 0.25 μm). Helium was employed as the carrier gas at a flow rate of 1.0 mL/min. For compound quantification, relative quantification was performed based on peak area normalization, and results are presented as relative contents (%). Quality control (QC) samples were analyzed every 10 injections, and compounds with RSD >30% were excluded.

### 2.6 Determination of composition and content of non-volatile metabolic compounds in Tibetan sheep muscle

A 50 mg sample of the longissimus dorsi muscle was added to a pre-chilled methanol/acetonitrile/water solution (2:2:1, v/v/v). The mixture was vortexed, sonicated for 30 min, and then allowed to stand at −20 °C for 10 min. The sample was subsequently centrifuged at 14,000 × g at 4 °C for 20 min. The supernatant was removed and subjected to vacuum drying. For mass spectrometry analysis, the dried residue was resuspended in 100 μL acetonitrile-water solution (acetonitrile:water = 1:1, v/v), vortexed, centrifuged at 14,000 × g at 4 °C for 15 min, and the supernatant was injected for analysis. LC-MS analysis was performed using an Agilent 1290 Infinity UHPLC system and an AB Triple TOF 6600 mass spectrometer. QC samples were prepared by mixing 10 μL of each sample and were analyzed every 9 injections to monitor instrumental stability and reproducibility. Acceptance criteria required that >90% of detected ion peaks in QC samples had a relative standard deviation (RSD) <30%. Raw data were converted to mzXML format using ProteoWizard, followed by alignment. Retention times were corrected prior to peak area extraction via XCMS. Only variables with non-zero values in >50% of samples within at least one group were retained. After normalizing total peak intensity, processed data were imported into SIMCA-P (version 14.1, Umetrics) to identify differential metabolites, followed by KEGG pathway analysis. The KEGG global metabolic pathway database (all metabolites in the KEGG database) was used as the background set for pathway annotation and enrichment analysis of the identified differential metabolites. The Benjamini–Hochberg (BH) method was applied to correct *p*-values for multiple testing, and pathways with a false discovery rate (FDR) <0.05 were considered significantly enriched. The top 20 most significantly enriched pathways (ranked by *p*-value) are presented. The KEGG result table includes both raw *p*-values and BH-corrected FDR values.

### 2.7 Statistical data analysis

Upon preliminary organization of the experimental data, one-way ANOVA was conducted on conventional meat quality data using SPSS software version 27.0, followed by Duncan’s multiple comparison test. The final results are expressed as mean ± standard deviation, with differences considered statistically significant at *p* < 0.05 and non-significant at *p* > 0.05.

## 3 Results

### 3.1 Effects of PMS addition on carcass quality of Tibetan sheep meat

Table 2 demonstrates that the incorporation of PMS did not significantly influence carcass weight, dressing percentage, height, chest circumference, slant length, eye muscle area, or rib meat thickness in Tibetan sheep (*p* > 0.05). Notably, Group S3 exhibited a significantly greater live weight compared to Groups K, S1, and S2 (*p* < 0.05). Furthermore, the abdominal wall thickness in Group S3 was significantly increased by 13.13 and 25.30% relative to Groups K and S1, respectively. The backfat thickness in Group S3 was also significantly higher than that of the other three groups, with increases of 7.41, 47.02, and 18.55%, respectively (*p* < 0.05). Direct comparison between S2 and S3 revealed that S3 had significantly greater abdominal wall thickness and backfat thickness (*p* < 0.05) compared to S2, confirming that 0.30% PMS is more effective in promoting fat deposition. This indicates that PMS can significantly promote the deposition of abdominal and back fat, thereby improving carcass fatness. In terms of average daily feed intake (ADFI), all PMS-added groups were significantly higher than the control group (*p* < 0.05). Regarding feed conversion ratio (FCR), the FCR of the S1 group (0.20% PMS) was significantly lower than that of the control group (*p* < 0.05), indicating the highest feed utilization efficiency. Adding PMS can improve the growth performance of Tibetan sheep by increasing feed intake and feed conversion efficiency. Taken together, although the addition of PMS has limited effects on basic carcass indicators, it can enhance the carcass quality of Tibetan Sheep by increasing live weight and fat deposition. Specifically, 0.30% PMS is optimal for improving carcass fatness, while 0.25% PMS is more effective for improving meat quality attributes.

**Table 2:** Effect of different levels of PMS on carcass quality of Tibetan sheep.

Project	Group				*p*-value
	K	S1	S2	S3	
Body height/cm	65.32 ± 2.50	68.47 ± 2.51	72.58 ± 7.39	67.38 ± 2.57	0.086
Bust/cm	83.93 ± 2.77	81.42 ± 3.90	83.92 ± 3.32	80.83 ± 3.00	0.320
Slant length/cm	72.92 ± 2.76	74.92 ± 3.55	75.33 ± 4.78	76.43 ± 3.23	0.505
Live weight/kg	34.68 ± 0.85^b^	35.37 ± 0.66^b^	35.43 ± 0.48^b^	36.34 ± 0.58^a^	0.007
Carcass weight/kg	16.52 ± 0.52	15.77 ± 0.84	16.03 ± 0.51	16.78 ± 0.4	0.055
Slaughter rate/%	47.62 ± 0.62	44.56 ± 1.74	45.25 ± 1.13	46.10 ± 0.65	0.060
Eye muscle area/cm^2^	21.33 ± 3.20	16.50 ± 2.99	21.00 ± 2.24	22.50 ± 2.75	0.080
Rib thickness/mm	25.33 ± 0.66	22.25 ± 1.35	23.78 ± 1.89	26.11 ± 2.12	0.070
Abdominal wall thickness/mm	20.80 ± 1.30^bc^	18.78 ± 1.51^c^	22.09 ± 1.61^ab^	23.53 ± 1.07^a^	<0.01
Backfat thickness/mm	20.23 ± 1.14^b^	14.78 ± 1.68^c^	18.33 ± 1.24^b^	21.73 ± 1.99^a^	<0.01
ADG/(kg/d)	0.21 ± 0.02	0.23 ± 0.01	0.22 ± 0.01	0.23 ± 0.01	0.066
FCR	6.38 ± 0.49^a^	5.68 ± 0.30^b^	6.04 ± 0.38^ab^	5.99 ± 0.36^ab^	0.037
ADFI/(kg/d)	1.29 ± 0.33^c^	1.35 ± 0.31^b^	1.39 ± 0.33^a^	1.35 ± 0.30^b^	<0.01

ADG refers to average daily gain, FCR refers to feed conversion ratio and ADFI refers to the average daily feed intake. Different letters indicate significant differences between groups after dietary supplementation with different levels of PMS (*p* < 0.05). Group K was fed a basal diet without PMS, Group S1 was fed a diet supplemented with 0.20% PMS, Group S2 was fed a diet supplemented with 0.25% PMS, and Group S3 was fed a diet supplemented with 0.30% PMS.

### 3.2 Effects of PMS supplementation on edible quality and nutritional properties of Tibetan sheep longissimus dorsi muscle

An analysis of the edible quality and nutritional properties of Tibetan sheep meat supplemented with varying levels of PMS indicated that PMS supplementation did not have a significant impact on L^*^, cooking loss, thawing loss, or specific texture parameters, including hardness, viscosity, elasticity, chewiness, and cohesiveness (*p* > 0.05). The pH values of Tibetan sheep meat in Groups S2 and S3 at 45 min post-mortem were significantly lower compared to those in Groups K and S1 (*p* < 0.05). Following 24 h of aging, the pH24h value in Group S2 was significantly elevated relative to the other three groups (*p* < 0.05), and the rate of pH decline in Groups S2 and S3 was comparatively slower. In terms of colorimetric analysis, the a^*^ value for Group S2 was significantly higher than that of Groups K and S3 (*p* < 0.05), whereas the b^*^ value was significantly lower than those of the other three groups. Concerning meat water-holding capacity, Groups S1, S2, and S3 exhibited significantly higher water-holding capacities than Group K (*p* < 0.05), and the shear force measurements for Groups S1 and S2 were significantly lower than those for Group K (*p* < 0.05). Direct comparison between S2 and S3 showed no significant difference in water-holding capacity (*p* > 0.05), but S2 exhibited a trend toward lower shear force, indicating better tenderness.

Moisture, crude protein, and crude fat are considered conventional nutritional components of muscle tissue. As indicated in Table 3, PMS supplementation did not exert a significant effect on the moisture and protein content in the longissimus dorsi muscle (*p* > 0.05). However, the fat content in Group S2 was significantly greater than that in Groups K, S1, and S3 (*p* < 0.05). A comprehensive comparison of the four groups indicates that Tibetan sheep meat from the S2 group exhibits greater advantages in both edible quality and nutritional quality.

**Table 3:** Effect of PMS addition on the edible and nutritional quality of the longissimus dorsi muscle of Tibetan sheep.

Project	Group				*p*-value
	K	S1	S2	S3	
Edible quality					
pH45min	7.10 ± 0.02^a^	6.96 ± 0.06^ab^	6.88 ± 0.03^b^	6.69 ± 0.04^c^	0.001
pH24h	5.75 ± 0.02^c^	5.94 ± 0.03^b^	6.18 ± 0.04^a^	6.12 ± 0.03^a^	<0.01
*L* ^*^	46.35 ± 2.11	43.10 ± 0.87	43.04 ± 0.40	43.84 ± 0.30	0.134
*a* ^*^	19.93 ± 0.78^b^	23.45 ± 1.04^ab^	31.37 ± 1.75^a^	16.83 ± 0.96^b^	0.008
*b* ^*^	42.11 ± 1.84^a^	18.57 ± 1.18^b^	18.69 ± 1.22^b^	23.60 ± 1.28^b^	<0.01
Cooking loss rate/%	31.48 ± 2.65	35.43 ± 2.41	35.92 ± 3.21	32.86 ± 2.68	0.058
Thaw loss/%	6.66 ± 1.16	5.19 ± 1.90	4.47 ± 1.06	4.89 ± 1.54	0.128
System hydraulic force/%	20.8 ± 0.35^b^	31.4 ± 1.00^a^	31.5 ± 0.44^a^	32.7 ± ±0.62^a^	0.032
Shear force/N	21.84 ± 4.98^b^	23.34 ± 4.24^b^	23.40 ± 7.93^ab^	26.68 ± 5.81^a^	0.043
Hardness/g	1058.67 ± 306	1101 ± 251.44	1818 ± 380.83	1465.83 ± 181.04	0.081
Viscosity/mJ	1.01 ± 0.65	1.14 ± 0.40	1.1.1 ± 0.61	1.07 ± 0.34	0.053
Elasticity/mm	3.39 ± 1.08	3.27 ± 0.58	2.93 ± 0.66	2.87 ± 0.50	0.179
Chewiness/mJ	16.06 ± 5.83	18.42 ± 4.33	16.9 ± 4.07	11.18 ± 6.72	0.128
Cohesion	0.55 ± 0.12	0.49 ± 0.11	0.46 ± 0.08	0.45 ± 0.10	0.056
Nutritional quality					
Moisture (%)	70.01 ± 3.43	69.10 ± 4.45	66.65 ± 4.12	70.08 ± 1.12	0.176
Fat (%)	2.93 ± 1.77^b^	2.67 ± 0.47^b^	10.83 ± 0.74^a^	6.57 ± 2.87^b^	0.005
Protein (%)	21.83 ± 1.03	22.70 ± 0.99	18.03 ± 2.05	20.90 ± 2.06	0.087

Different letters indicate significant differences (*p* < 0.05) between groups after adding different levels of PMS to the feed. L^*^, a^*^, and b^*^ represent the brightness, redness, and yellowing of the meat, respectively. Group K was fed with feed without PMS, Group S1 was fed with feed with 0.20% PMS, Group S2 was fed with feed with 0.25% PMS, and Group S3 was fed with feed with 0.30% PMS.

### 3.3 Effect of PMS supplementation on mineral element content in Tibetan sheep

The analysis of mineral element content in the longissimus dorsi muscle of Tibetan sheep subjected to varying levels of PMS feed is presented in Table 4. Statistically significant differences (*p* < 0.05) were identified among the four groups in the concentrations of six elements: Li, Ca, V, Cr, Fe, and Sn. Conversely, no significant differences (*p* > 0.05) were detected in the concentrations of 16 other elements, including B, Al, P, K, Ti, Mn, Ni, Cu, Zn, Se, Rb, Sr, Mo, Sn, Bn, Bi, and Mg. Among these elements, macronutrients such as K, P, Mg, Zn, Fe, and Ca were found in the highest concentrations across all groups, whereas the remaining elements were present in lower concentrations. Specifically, the Li content in Group K was significantly higher than that in the other three groups, while the contents of Ca, V, and Cr were significantly lower than those in Groups S2 and S3. The Fe content in Group S2 was the highest, significantly higher than that in Groups K and S3. This indicates that appropriate supplementation of PMS can improve the deposition of some key mineral elements in muscle to a certain extent, thereby enhancing the nutritional value of the meat.

**Table 4:** Effect of PMS addition on the mineral element content of the longissimus dorsi muscle of Tibetan sheep.

Project	Group				*p*-value
	K	S1	S2	S3	
Li	0.02 ± 0.01^b^	0.07 ± 0.02^a^	0.06 ± 0.01^a^	0.01 ± 0.01^b^	0.010
B	9.02 ± 0.20^a^	3.97 ± 0.38^c^	6.82 ± 0.26^a^	5.88 ± 1.22^b^	<0.001
Al	4.22 ± 1.02	4.80 ± 3.93	6.23 ± 0.89	3.44 ± 2.07	0.681
P	2474.39 ± 307.60	2868.57 ± 541.06	2783.01 ± 183.65	2855.74 ± 256.26	0.656
K	9012.84 ± 715.22	9519.95 ± 818.35	9510.52 ± 435.28	9424.80 ± 774.29	0.870
Ca	31.72 ± 5.58^c^	80.11 ± 8.17^a^	58.27 ± 9.66^b^	57.35 ± 2.67^b^	0.007
Ti	0.77 ± 0.27	0.73 ± 0.48	0.71 ± 0.21	0.55 ± 0.42	0.920
V	0.26 ± 0.04^a^	0.13 ± 0.03^c^	0.21 ± 0.00^ab^	0.19 ± 0.02^b^	0.010
Cr	3.48 ± 0.75^a^	0.94 ± 0.57^b^	0.87 ± 0.24^b^	0.71 ± 0.33^b^	0.002
Mn	0.42 ± 0.14	0.84 ± 0.24	0.86 ± 0.06	0.45 ± 0.28	0.099
Fe	35.07 ± 1.04^b^	59.33 ± 5.93^a^	61.03 ± 5.73^a^	44.30 ± 13.59^ab^	0.032
Ni	0.67 ± 0.08	0.25 ± 0.18	0.14 ± 0.09	0.55 ± 0.34	0.093
Cu	2.89 ± 0.35	3.44 ± 0.46	3.62 ± 0.59	3.34 ± 0.88	0.681
Zn	37.66 ± 9.05	40.26 ± 10.42	29.43 ± 2.56	30.60 ± 2.50	0.403
Se	0.29 ± 0.04	0.24 ± 0.02	0.25 ± 0.03	0.25 ± 0.02	0.441
Rb	14.86 ± 0.73	17.87 ± 3.15	15.38 ± 0.88	15.35 ± 1.04	0.370
Sr	0.18 ± 0.04	0.29 ± 0.12	0.33 ± 0.03	0.17 ± 0.11	0.199
Mo	1.56 ± 0.60	5.22 ± 3.35	8.33 ± 0.80	7.98 ± 3.57	0.087
Sn	1.45 ± 0.07^c^	2.02 ± 0.04^a^	1.80 ± 0.04^ab^	1.70 ± 0.17^b^	0.003
Ba	0.15 ± 0.06	0.32 ± 0.26	0.27 ± 0.08	0.50 ± 0.39	0.541
Bi	0.64 ± 0.01^a^	0.30 ± 0.09^b^	0.58 ± 0.01^b^	0.62 ± 0.02^b^	<0.01
Mg	387.82 ± 45.46	471.15 ± 80.49	456.15 ± 21.41	460.51 ± 43.22	0.780

Different letters indicate significant differences between groups after adding different levels of PMS to the feed (*p* < 0.05). Group K was fed with feed without PMS, Group S1 was fed with feed with 0.20% PMS, Group S2 was fed with feed with 0.25% PMS, and Group S3 was fed with feed with 0.30% PMS.

### 3.4 Effects of PMS addition on sensory quality of Tibetan sheep

**Figure 1:**
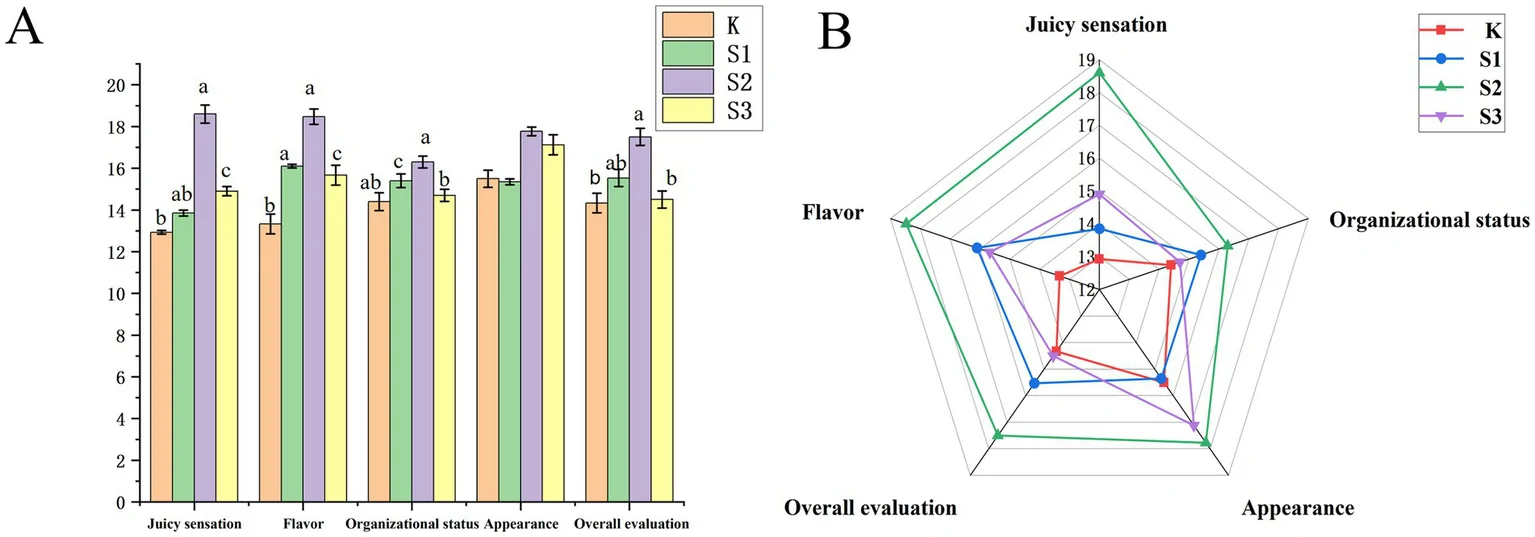
Sensory rating chart and radar chart. **(A)** The bar chart displays the sensory attribute ratings (mean ± standard error) for K, S1, S2, and S3. The different lowercase letters above the bar indicate significant differences (*p* < 0.05) between groups within the same attribute. **(B)** The radar chart displays the comprehensive sensory performance of the sample in five attributes (juiciness, flavor, organizational structure, appearance, and overall evaluation). Group K was fed with feed without PMS, Group S1 was fed with feed with 0.20% PMS, Group S2 was fed with feed with 0.25% PMS, and Group S3 was fed with feed with 0.30% PMS.

As shown in graphical abstract, the sensory quality analysis of Tibetan sheep meat showed that the addition of different levels of had a significant impact characteristics had a significant impact on sensory indicators such as juiciness, flavor, tissue structure, and overall evaluation; The bar chart in Figure 1A quantifies the differences in scores among groups, while the radar chart in Figure 1B intuitively presents the comprehensive contour of sensory quality. Specifically, the scores of S2 group in the three core indicators of juiciness, flavor, and tissue structure were significantly better than those of K group and S1 group (*p* < 0.05), and also higher than those of S3 group; In terms of overall evaluation, the S2 group scored significantly higher than the S3 group (*p* < 0.05), while there was no significant difference in appearance indicators between the groups. The radar chart further confirms this trend, with S2 group showing the most outstanding comprehensive sensory performance in juiciness, flavor, organizational structure, and overall evaluation.

### 3.5 Effects of PMS supplementation on amino acid content and composition in Tibetan sheep

Table 5 presents the effect of different levels of PMS supplementation on the amino acid composition and content of the longissimus dorsi muscle in Tibetan sheep. Statistical analysis showed that there were no significant differences (*p* > 0.05) in total amino acid (TAA), essential amino acid (EAA), or non-essential amino acid (NEAA) contents among the four experimental groups. Notably, the alanine concentration in Group S2 was significantly higher than that in the other three groups, whereas the citrulline content in Group K was significantly greater than that in Groups S2 and S3 (*p* < 0.05).

**Table 5:** Effect of PMS addition on the amino acid content of the longissimus dorsi muscle of Tibetan sheep.

Project	Group				*p*-value
	K	S1	S2	S3	
Alanine	3711.96 ± 46.58^b^	3581.58 ± 176.18^b^	3877.33 ± 129.69^a^	3455.49 ± 65.04^b^	0.034
Aminoadipic acid	28.17 ± 3.58	34.42 ± 2.99	33.05 ± 4.09	31.01 ± 1.67	0.304
Arginine	796.74 ± 53.20	903.53 ± 74.83	1025.97 ± 107.09	896.31 ± 69.39	0.105
Asparagine	208.99 ± 9.28	207.42 ± 12.93	216.70 ± 12.08	195.92 ± 25.61	0.657
Aspartic acid	175.11 ± 61.26	174.30 ± 35.48	294.07 ± 92.94	260.28 ± 111.36	0.392
Citrulline	391.40 ± 77.18^a^	328.90 ± 54.57^ab^	254.30 ± 11.50^bc^	207.88 ± 16.45^c^	0.023
Creatine	4000.11 ± 216.36	4096.96 ± 144.36	4180.74 ± 155.47	3919.79 ± 59.68	0.410
Cysteine	0.56 ± 0.25	0.12 ± 0.03	0.10 ± 0.05	0.35 ± 0.31	0.147
Glutamate	295.85 ± 71.93	256.92 ± 37.03	264.44 ± 26.58	248.14 ± 30.38	0.742
Glutamine	261.84 ± 56.40	324.08 ± 45.49	373.95 ± 86.15	482.73 ± 131.95	0.152
Glycine	1298.25 ± 288.84	1502.92 ± 510.21	1552.91 ± 371.90	1480.33 ± 404.35	0.925
Histidine	873.06 ± 79.30	873.78 ± 34.78	896.52 ± 121.38	987.21 ± 108.35	0.583
Hydroxyproline	80.29 ± 18.87	67.10 ± 16.68	94.00 ± 41.78	58.80 ± 1.16	0.528
Ornithine	15.71 ± 1.85	16.10 ± 9.34	12.75 ± 5.58	11.59 ± 1.20	0.813
Proline	72.55 ± 9.42	71.86 ± 5.29	75.92 ± 11.38	62.99 ± 8.89	0.552
Serine	308.86 ± 41.48	323.63 ± 32.47	302.02 ± 60.37	299.07 ± 32.54	0.941
Aminoethanesulfonic acid	2938.42 ± 170.98	3443.27 ± 533.84	2683.08 ± 1058.54	2910.31 ± 807.27	0.761
Tyrosine	65.60 ± 19.46	78.41 ± 7.72	56.97 ± 5.53	73.44 ± 14.58	0.428
Isoleucine	121.89 ± 31.96	135.20 ± 1.94	132.19 ± 9.92	133.31 ± 9.49	0.870
leucine	445.90 ± 78.92	479.96 ± 15.54	482.27 ± 16.06	454.18 ± 22.68	0.777
Lysine	138.33 ± 51.42	142.89 ± 10.98	128.95 ± 9.09	156.70 ± 50.98	0.896
Methionine	50.79 ± 21.00	58.67 ± 5.38	47.92 ± 2.39	56.90 ± 3.43	0.774
Phenylalanine	131.80 ± 27.57	138.81 ± 5.87	137.70 ± 9.80	136.55 ± 10.52	0.971
Threonine	241.61 ± 25.16	267.52 ± 53.04	259.84 ± 38.89	244.64 ± 69.06	0.943
Tryptophan	162.12 ± 45.85	179.83 ± 6.78	187.02 ± 19.71	215.91 ± 31.87	0.394
Valine	338.30 ± 48.69	348.75 ± 30.65	339.38 ± 20.73	307.83 ± 17.29	0.618
TAA	17704.51 ± 1556.79	18036.93 ± 1864.4	17570.71 ± 2407.96	16979.83 ± 2088.20	0.655
EAA	1521.04 ± 330.57	1751.63 ± 130.18	1375.89 ± 105.86	1398.19 ± 198.03	0.211
NEAA	15523.47 ± 1226.22	16285.3 ± 1734.2	16194.82 ± 2302.1	15581.64 ± 1890.2	0.552

Different letters indicate significant differences between groups after adding different levels of PMS to the feed (*p* < 0.05); TAA refers to total amino acids, EAA refers to essential amino acids, and NEAA refers to non essential amino acids. Group K was fed with feed without PMS, Group S1 was fed with feed with 0.20% PMS, Group S2 was fed with feed with 0.25% PMS, and Group S3 was fed with feed with 0.30% PMS.

### 3.6 Effects of PMS supplementation on fatty acid content and composition in the longissimus dorsi muscle of Tibetan sheep

Table 6 presents the effects of dietary supplementation with different levels of PMS on the fatty acid composition and content in the longissimus dorsi muscle of Tibetan sheep. A total of 36 fatty acids were detected across the four groups. The total content of saturated fatty acids (SFAs) did not differ significantly among the groups (*p* > 0.05), whereas the total contents of monounsaturated fatty acids (MUFAs) and polyunsaturated fatty acids (PUFAs) were significantly higher in Groups S2 and S3 than in Group K (*p* < 0.05). Among SFAs, palmitic acid (C16:0) and stearic acid (C18:0) were the most abundant, and their concentrations in Group S3 were significantly higher than those in Group K (*p* < 0.05). For MUFAs, palmitoleic acid (C16:1) and oleic acid (C18:1) were predominant, and the content of palmitoleic acid in Groups S1, S2, and S3 was significantly higher than that in Group K (*p* < 0.05). Within PUFAs, linoleic acid (C18:2n-6) and linolenic acid (C18:3n-3) were the most abundant, with notably higher levels observed in Group S2. Furthermore, the PUFAs closely associated with human health-eicosapentaenoic acid (EPA), docosahexaenoic acid (DHA), arachidonic acid (ARA), and docosapentaenoic acid (DPA)—were present at the highest concentrations in Group S2. Collectively, these findings indicate that appropriate supplementation with PMS can effectively enhance the deposition of beneficial fatty acids in Tibetan sheep meat, particularly PUFAs that are beneficial to human health.

**Table 6:** Effect of PMS addition on fatty acid content of longissimus dorsi muscle in Tibetan sheep.

Project	Group				*p*-value
	K	S1	S2	S3	
C6:0	0.05 ± 0.01	0.04 ± 0.00	0.05 ± 0.00	0.05 ± 0.01	0.916
C8:0	0.38 ± 0.06	0.27 ± 0.00	0.37 ± 0.07	0.48 ± 0.14	0.358
C10:0	5.66 ± 0.40	9.87 ± 2.84	4.50 ± 0.82	5.98 ± 0.41	0.121
C11:0	0.14 ± 0.04	0.21 ± 0.01	0.13 ± 0.04	0.26 ± 0.05	0.052
C12:0	7.38 ± 2.33^c^	14.61 ± 1.29^a^	7.79 ± 1.65^b^	26.45 ± 2.14^a^	0.001
C13:0	0.46 ± 0.23^a^	0.55 ± 0.05^a^	0.28 ± 0.06^b^	1.04 ± 0.11^a^	0.015
C14:0	135.31 ± 54.61^c^	338.25 ± 7.06^a^	178.39 ± 23.10^b^	483.17 ± 1.21^a^	0.004
C15:0	11.24 ± 3.12^c^	21.16 ± 0.49^a^	11.94 ± 2.05^b^	38.21 ± 0.64^a^	0.002
C16:0	1614.21 ± 348.04^c^	2709.22 ± 283.83^ab^	2066.16 ± 214.3^bc^	3398.88 ± 104.1^a^	0.007
C17:0	38.37 ± 7.84^b^	74.93 ± 11.13^a^	46.69 ± 10.31^a^	129.07 ± 4.31^a^	<0.01
C18:0	1161.44 ± 103.06^b^	1062.94 ± 263.63^b^	1331.76 ± 132.75^b^	2611.68 ± 32.80^a^	<0.01
C20:0	3.25 ± 0.93	5.50 ± 2.37	7.45 ± 2.83	13.08 ± 0.26	0.066
C21:0	0.67 ± 0.07	0.53 ± 0.10	0.58 ± 0.00	0.83 ± 0.07	0.066
C22:0	0.59 ± 0.08^b^	0.28 ± 0.07^c^	0.67 ± 0.18^ab^	0.92 ± 0.14^a^	0.006
C23:0	0.13 ± 0.01^ab^	0.06 ± 0.02^c^	0.11 ± 0.01^bc^	0.19 ± 0.03^a^	0.011
C24:0	0.23 ± 0.02^b^	0.09 ± 0.03^b^	0.22 ± 0.06^b^	0.32 ± 0.06^a^	0.023
SFAs	2979.51 ± 520.85	4238.51 ± 572.92	3657.09 ± 388.23	6710.61 ± 148.48	0.145
C14:1N-5	4.60 ± 2.26^c^	11.91 ± 1.78^ab^	6.53 ± 0.48^bc^	15.62 ± 0.11^a^	0.018
C15:1n5 cis	0.18 ± 0.00	0.14 ± 0.02	0.61 ± 0.08	0.34 ± 0.27	0.081
C16:1N-7 cis	151.11 ± 49.55^b^	318.09 ± 11.39^a^	207.81 ± 21.38^b^	317.71 ± 1.63^b^	0.029
C17:1N-7 cis	43.81 ± 12.14^b^	58.47 ± 14.28^ab^	56.09 ± 15.09^b^	87.43 ± 8.88^a^	0.048
C18:1N-9T	27.20 ± 10.89	28.08 ± 8.62	37.63 ± 18.27	45.34 ± 10.82	0.474
C18:1N-9	3649.17 ± 1012.48	3863.95 ± 960.16	4288.62 ± 782.59	5755.37 ± 36.58	0.108
C20:1N-9 cis	17.98 ± 3.00^b^	15.21 ± 3.19^b^	17.08 ± 1.92^b^	27.49 ± 3.42^a^	0.013
C22:1N-9	1.38 ± 0.65	2.52 ± 0.58	2.84 ± 1.52	3.99 ± 0.86	0.142
C24:1N-9 cis	2.11 ± 0.68	1.63 ± 0.52	2.08 ± 0.38	1.36 ± 0.15	0.378
MUFAs	3690.63 ± 101.65^b^	4,300 ± 100.52^ab^	4619.29 ± 141.71^a^	6254.22 ± 62.72^a^	<0.01
C18:3N-3,6,9 all-cis	58.91 ± 11.03	50.51 ± 4.24	62.65 ± 17.49	69.71 ± 12.01	0.497
C18:2N-6,9 all-trans	11.87 ± 3.64	11.01 ± 2.71	14.11 ± 5.26	18.29 ± 2.25	0.266
C18:2N-6,9 all-cis	755.55 ± 11.00	609.02 ± 51.57	745.39 ± 135.73	710.54 ± 161.85	0.550
C18:2 N-6,9,12 all-cis	8.63 ± 0.30^b^	6.72 ± 0.65^b^	9.42 ± 0.97^b^	9.99 ± 0.43^a^	0.005
C20:2N-6,9 all-cis	43.53 ± 5.97	40.16 ± 13.76	45.48 ± 4.30	48.78 ± 5.49	0.770
C20:3N-6,9,12 all-cis	23.31 ± 2.17	22.39 ± 2.21	24.93 ± 2.46	21.81 ± 1.67	0.527
C22:4 N-6,9,12,15 cis	17.37 ± 3.65	16.16 ± 1.82	18.72 ± 0.50	16.96 ± 2.46	0.758
Total_N6	860.26 ± 26.73^ab^	705.4 ± 72.72^b^	862.05 ± 149.22^a^	826.37 ± 174.15^a^	0.044
ARA	341.70 ± 37.69	265.87 ± 29.15	338.84 ± 23.15	300.83 ± 41.31	0.156
EPA	34.19 ± 4.42	30.85 ± 2.94	40.73 ± 4.53	32.74 ± 4.25	0.165
DPA	65.15 ± 4.66	63.64 ± 7.80	79.06 ± 1.66	68.78 ± 6.31	0.090
DHA	11.85 ± 1.99	10.23 ± 1.92	11.91 ± 1.45	9.29 ± 0.81	0.349
PUFAs	1372.05 ± 86.52^b^	1126.5 ± 118.77^b^	1395.24 ± 346.72^a^	1307.72 ± 238.84^b^	0.007

Different letters indicate significant differences between groups after adding different levels of PMS to the feed (*p* < 0.05); SFAs, total fatty acids; MUFAs, monounsaturated fatty acids; total_PUFA, polyunsaturated fatty acids; total: N3, omega-3 polyunsaturated fatty acids; total: N6, omega-6 polyunsaturated fatty acids. Group K was fed with feed without PMS, Group S1 was fed with feed with 0.20% PMS, Group S2 was fed with feed with 0.25% PMS, and Group S3 was fed with feed with 0.30% PMS.

### 3.7 Effect of PMS addition on volatile flavor metabolites in Tibetan sheep longissimus dorsi muscle

#### 3.7.1 Quality control analysis

**Figure 2:**
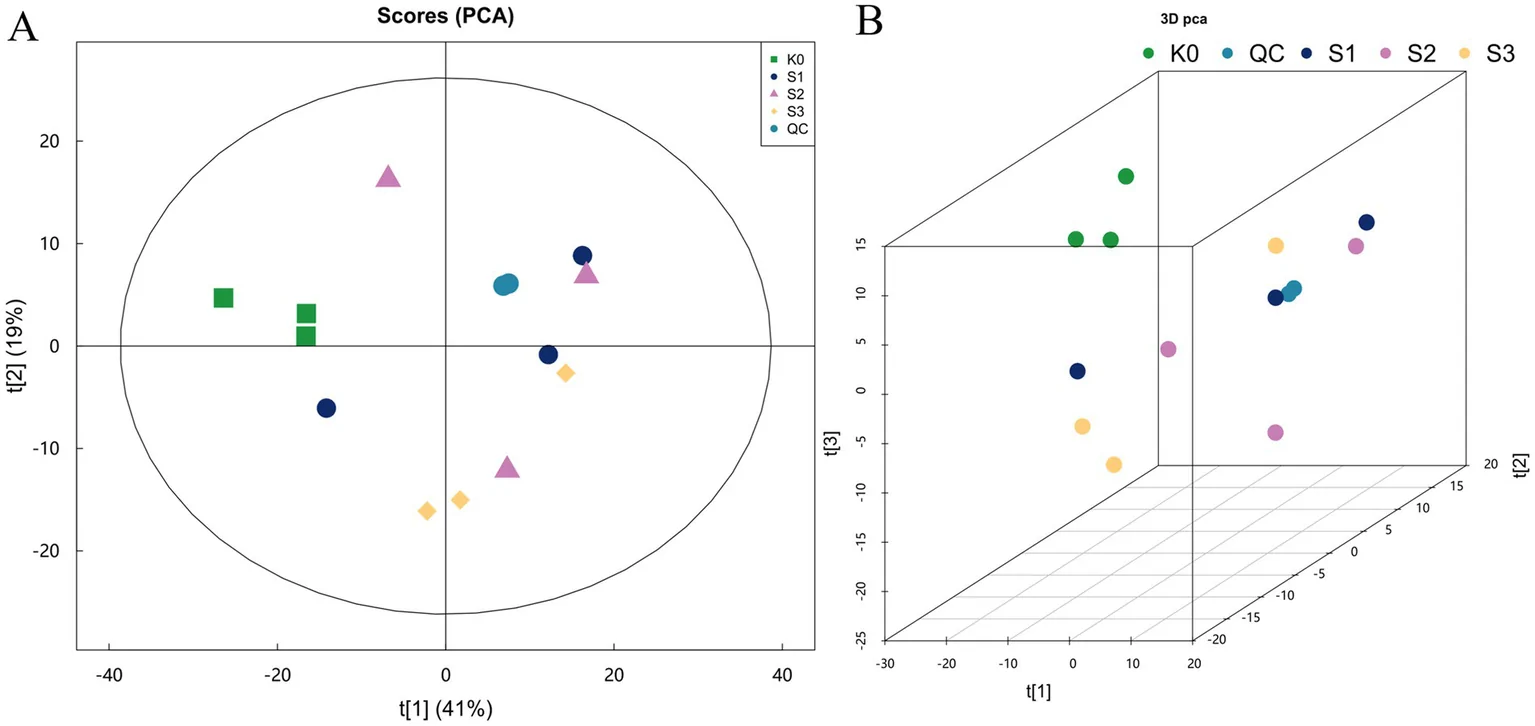
PCA score plot of volatile compounds in Tibetan lamb meat with different PMS added. **(A)** Two-dimensional PCA score plot showing the separation of samples K, S1, S2, S3, and QC. The first principal component (*t*[1]) explains 41% of the total variance, while the second principal component (*t*[2]) explains 19%. The ellipse represents the 95% confidence interval. **(B)** Three-dimensional PCA score plot illustrating the distribution of samples in the space defined by *t*[1], *t*[2], and *t*[3]. Each sample group is represented by a distinct color and symbol as indicated in the legend. Group K was fed with feed without PMS, Group S1 was fed with feed with 0.20% PMS, Group S2 was fed with feed with 0.25% PMS, and Group S3 was fed with feed with 0.30% PMS.

**Figure 3:**
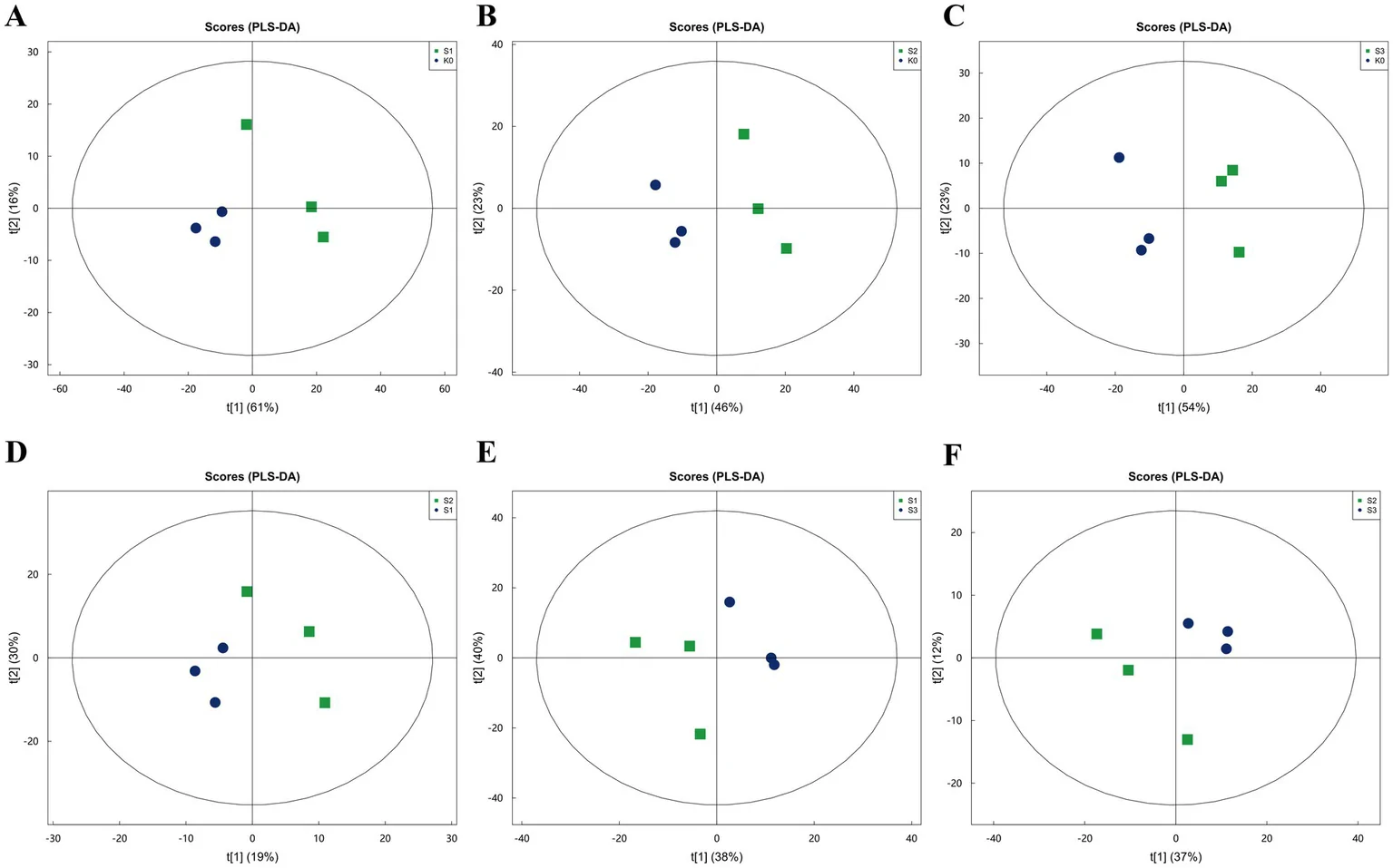
PLS-DA plots of volatile compounds in Tibetan lamb meat with different PMS added **(A–F)** (**A**: K and S1; **B**: K and S2; **C**: K and S3; **D**: S1 and S2; **E**: S1 and S3; **F**: S2 and S3). Two-dimensional PLS-DA score plots showing the separation of two sample groups across different comparisons. The first principal component (*t*[1]) and second principal component (*t*[2]) are indicated on the axes, with the percentage of explained variance shown in parentheses for each component. The ellipse represents the 95% confidence interval. Group K was fed with feed without PMS, Group S1 was fed with feed with 0.20% PMS, Group S2 was fed with feed with 0.25% PMS, and Group S3 was fed with feed with 0.30% PMS.

**Figure 4:**
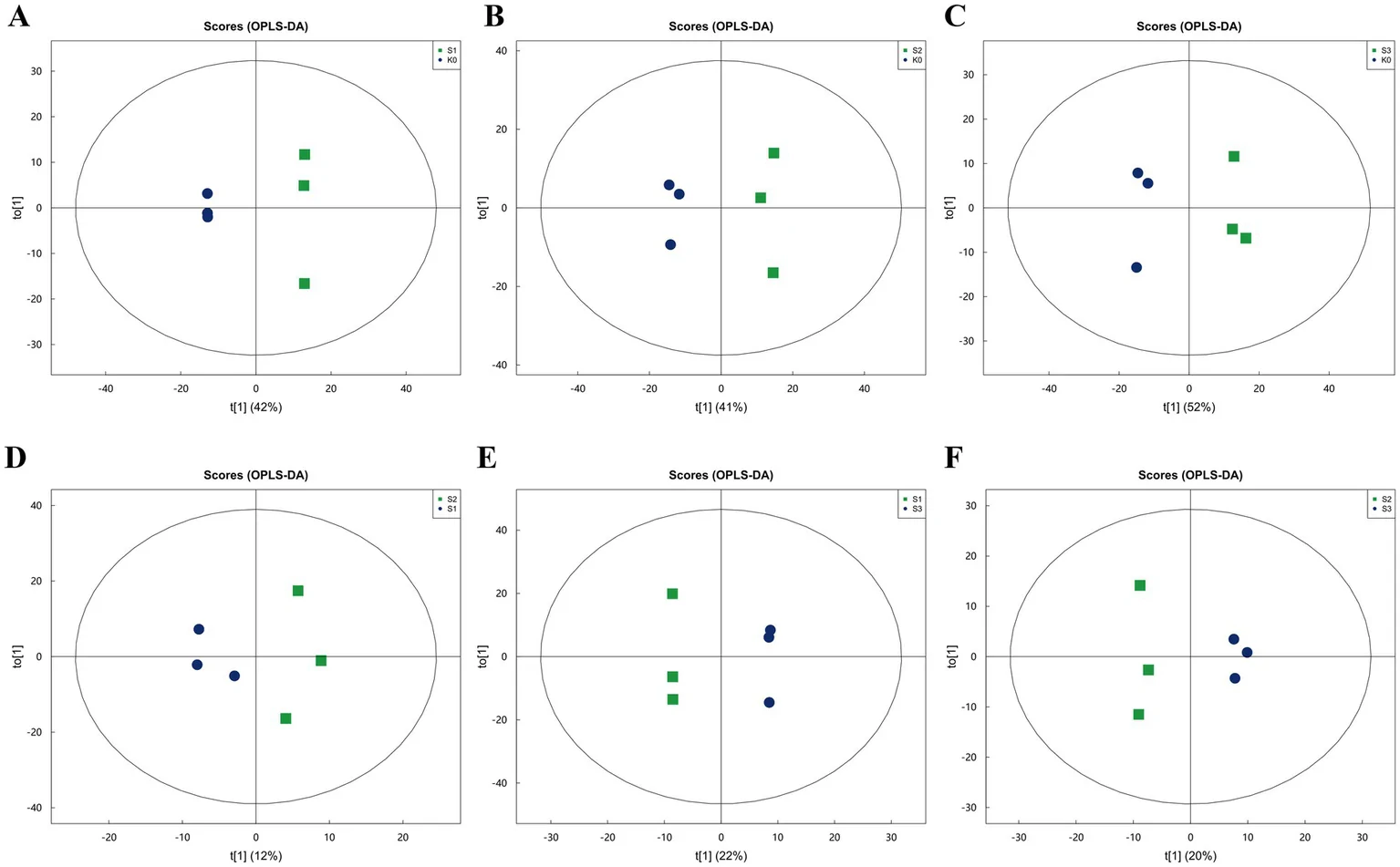
OPLS-DA plots **(A–F)** of volatile compounds in Tibetan lamb meat with different PMS added (**A**: K and S1; **B**: K and S2; **C**: K and S3; **D**: S1 and S2; **E**: S1 and S3; **F**: S2 and S3). **(A–F)** Two-dimensional OPLS-DA score plots showing the separation of two sample groups across different pairwise comparisons. The predictive component (*t*[1]) and orthogonal component (*t*[2]) are indicated on the axes, with the percentage of explained variance shown in parentheses for each component. The ellipse represents the 95% confidence interval. Group K was fed with feed without PMS, Group S1 was fed with feed with 0.20% PMS, Group S2 was fed with feed with 0.25% PMS, and Group S3 was fed with feed with 0.30% PMS.

To visually assess the global metabolic differences among the groups, principal component analysis (PCA) was conducted on the volatile compound profiles. As shown in Figure 2, the scores plot revealed a clear separation between the control group (K) and all treatment groups (S1, S2, and S3). The first principal component (t1), which explained 41% of the total variance, distinctly separated the control group from the treatment groups, indicating significant differences in volatile compound profiles between the K group and the treated groups (*p* < 0.05). Furthermore, the second principal component (t2), accounting for 19% of the variance, effectively distinguished the three treatment groups (S1, S2, and S3) from one another, highlighting the specific metabolic signatures associated with different supplementation levels of potassium magnesium sulfate. Additionally, partial least squares discriminant analysis (PLS-DA) and orthogonal partial least squares discriminant analysis (OPLS-DA) models were used to further analyze the metabolic differences among the four sample groups in Figures 3, 4, respectively. In the score plots of the two models, the horizontal axis *t*1 represents the first principal component, which represents the metabolic variance information that contributes the most to the inter group differences; The vertical axis *t*2 represents the second principal component, which reflects the suboptimal variance information and mainly assists in presenting the distribution characteristics within the group. From the A–F subgraphs of Figure 3 (PLS-DA) and Figure 4 (OPLS-DA), it can be seen that all samples within each group exhibit a tight clustering trend, while clear spatial separation is achieved between groups. As depicted in Supplementary Figure S1, *R*^2^*Y* represents the cumulative variance explained by the model, with higher values indicating greater explanatory power. *Q*^2^ denotes the proportion of data variance predicted by the model, where *Q*^2^ > 0.5 suggests a stable and reliable model, 0.3 < *Q*^2^ < 0.5 indicates good model stability, and *Q*^2^ < 0.3 suggests low model reliability. All models demonstrated acceptable predictive capability and stability. In short, the PLS-DA and OPLS-DA models demonstrated statistically significant intergroup separation of metabolites. These findings suggest that the incorporation of various PMS notably influenced the volatile metabolites in Tibetan sheep muscle.

#### 3.7.2 Qualitative and quantitative analysis of volatile metabolites in Tibetan sheep meat with PMS

To further elucidate the differences in volatile components of Tibetan sheep meat with PMS addition, GC-MS was utilized for the qualitative and quantitative analysis of volatile compounds. A total of 460 aromatic compounds were identified across the four groups, comprising 91 halogenated compounds, 69 alcohols, 56 alkenes, 54 alkanes, 46 nitrogen-containing compounds, 40 esters, 21 benzene ring compounds, 14 ethers, 14 ketones, 12 acids, 9 aldehydes, 9 alkyne compounds, 8 sulfur-containing compounds, 7 furan compounds, 5 epoxide compounds, 3 heterocyclic compounds, 1 peroxide compound, and 1 pyran compound. Among these, halogenated compounds, alcohols, alkanes, alkenes, nitrogen-containing compounds, and esters were identified as the primary volatile flavor compounds. Based on significance and content, 33 volatile compounds were selected (Table 7), including 8 esters, 6 aldehydes, 6 alcohols, and 6 acids. The total concentrations of esters, aldehydes, and acids exhibited an upward trend with increasing PMS dosage. Notably, compounds such as methyl 3-methylpentanoate, 2-ethylbutyl acetate, methyl heptanoate, 2-octanol, 1-hydrazinopropan-2-ol, 4-methylene-1,2-dimethylcyclopent-1-ene, and (2-methylenecyclopropyl) methanol were present in significantly high concentrations. Among these, methyl 3-methylpentanoate demonstrated the highest levels in Groups S2 and S3, contributing a fresh fruity aroma to the lamb. Methyl heptanoate attained its maximum concentration in Group S2, enhancing the fresh fruity aroma by 62.89% compared to the control group, in addition to imparting a fatty aroma and fruity notes. Simultaneously, compounds such as pentyl formate, decanal, (Z)-3-hexenal, 1-octanol, 2-methylheptanoic acid, and 6-methyl-2-heptanone also showed increased levels with rising PMS concentrations, thereby positively influencing the lamb’s flavor profile. In contrast, 1-hydrazinopropan-2-ol exhibited the lowest concentration in Group S2, with a decrease of 37.86% relative to the control group. Furthermore, 4-methylene-1,2-dimethylcyclopent-1-ene, and (2-methylenecyclopropyl) methanol, showed a significant decrease with increasing PMS addition (*p* < 0.05).

**Table 7:** Effects of PMS addition on volatile flavor metabolites in the longissimus dorsi muscle of Tibetan sheep.

Project	Aroma description	Group				*p*-value
		K	S1	S2	S3	
Esters		18.11 ± 1.10	25.5 ± 1.94	31.52 ± 2.21	31.37 ± 1.39	<0.01
Methyl 3-methylbutanoate	Fruity aroma	0.08 ± 0.03	0.20 ± 0.06	0.30 ± 0.10	0.33 ± 0.08	0.378
Methyl 3-methylpentanoate	Fruity aroma	9.02 ± 0.04	14.37 ± 0.45	18.69 ± 1.01	19.18 ± 0.21	0.031
Pentyl formate	Fruity and wine	0.29 ± 0.20	0.75 ± 0.32	1.01 ± 0.61	1.59 ± 0.30	0.430
(E)-Methyl 3-hexenoate	Herbaceous fragranc	0.01 ± 0.00	0.02 ± 0.00	0.02 ± 0.02	0.01 ± 0.02	0.705
2-Ethylbutyl acetate	Fruity and wine	5.38 ± 0.49	3.44 ± 0.40	3.02 ± 0.18	3.91 ± 0.15	0.128
Methyl heptanoate	Fruity aroma	2.90 ± 0.15	6.18 ± 0.45	7.81 ± 0.09	5.84 ± 0.53	0.021
Methyl 4-hexenoate	Herbaceous fragrance	0.03 ± 0.01	0.06 ± 0.03	0.12 ± 0.10	0.09 ± 0.02	0.622
Methyl 2-phenylacetate	Floral fragrance	0.40 ± 0.18	0.48 ± 0.23	0.52 ± 0.10	0.42 ± 0.08	0.796
Aldehydes		0.59 ± 0.30	1.01 ± 0.50	2.97 ± 0.46	2.04 ± 0.35	0.042
Decanal	Citrus, fat fragrance	0.05 ± 0.01	0.09 ± 0.06	0.11 ± 0.07	0.13 ± 0.03	0.396
3-Methylpentanal	Almond and nut fragrance	0.07 ± 0.03	0.36 ± 0.22	1.76 ± 0.12	0.90 ± 0.24	0.519
(Z)-3-Hexenal	Herbaceous fragrance	0.41 ± 0.24	0.46 ± 0.17	0.96 ± 0.20	0.94 ± 0.05	0.360
3-Methylhexanal	Nut fragrance	0.02 ± 0.01	0.04 ± 0.03	0.08 ± 0.05	0.01 ± 0.01	0.120
(E)-2-Hexenal	Grass and fruit fragrance	0.02 ± 0.01	0.02 ± 0.00	0.03 ± 0.01	0.02 ± 0.00	0.471
Nonanal	Fat, citrus flavor	0.02 ± 0.00	0.04 ± 0.02	0.03 ± 0.01	0.04 ± 0.01	0.183
Alcohols		69.99 ± 2.99	61.51 ± 3.70	53.40 ± 3.14	56.38 ± 2.91	0.032
2-Octanol	Mushroom flavor	37.18 ± 1.29	37.46 ± 1.52	31.62 ± 1.41	31.11 ± 1.34	0.391
1-Octanol	Mushroom, fat flavor	0.31 ± 0.11	0.72 ± 0.39	0.83 ± 0.40	1.15 ± 0.21	0.285
2-Methyl-1-pentanol	Grape wine fragrance	0.08 ± 0.06	0.12 ± 0.06	0.02 ± 0.02	0.05 ± 0.00	0.225
3,4-Dimethyl-1-pentanol	Mellow fragrance	0.08 ± 0.03	0.04 ± 0.03	0.01 ± 0.02	0.02 ± 0.01	0.023
Cis-p-Mentha-2,8-dien-1-ol	Menthol	0.01 ± 0.00	0.01 ± 0.00	0.02 ± 0.00	0.01 ± 0.00	0.032
1-Hydrazinopropan-2-ol	Pungent odor	32.33 ± 1.50	23.04 ± 1.70	20.90 ± 1.29	23.99 ± 1.35	0.141
Acids		3.01 ± 0.64	6.91 ± 0.38	7.48 ± 0.22	7.72 ± 0.86	0.421
L-Lactic acid	Sour milk flavor	0.08 ± 0.01	0.10 ± 0.03	0.13 ± 0.03	0.07 ± 0.02	0.155
2-Methylpentanoic acid	Cheese flavor	1.13 ± 0.60	3.35 ± 0.06	2.95 ± 0.08	4.29 ± 0.30	0.271
2-Methylheptanoic acid	Fatty taste	1.73 ± 0.02	3.49 ± 0.28	4.35 ± 0.11	3.32 ± 0.53	0.260
Heptanoic acid	Pungent odor	0.02 ± 0.00	0.02 ± 0.00	0.02 ± 0.00	0.02 ± 0.00	0.772
Nonanoic acid	Pungent odor	0.04 ± 0.01	0.03 ± 0.01	0.02 ± 0.00	0.02 ± 0.01	0.007
4-Methylnonanoic acid	Lanolin flavor	0.01 ± 0.00	0.01 ± 0.00	0.01 ± 0.00	0.00 ± 0.00	0.032
Oxygen-containing heterocyclic compounds		0.23 ± 0.04	0.28 ± 0.03	0.27 ± 0.06	0.25 ± 0.06	0.211
Acetic acid, (2-methoxyethoxy)	Faint sweet fragrance	0.19 ± 0.03	0.20 ± 0.01	0.20 ± 0.02	0.15 ± 0.04	0.902
2-Pentylfuran	Grass, butter fragrance	0.01 ± 0.00	0.05 ± 0.01	0.04 ± 0.02	0.07 ± 0.02	0.253
2(3H)-Furanone, dihydro-3-hydroxy-4,4-dimethy	Caramel, sweet	0.03 ± 0.01	0.03 ± 0.01	0.03 ± 0.02	0.03 ± 0.00	0.939
Ketones		0.47 ± 0.15	0.68 ± 0.09	1.00 ± 0.04	0.68 ± 0.04	0.231
2-Methyl-3-pentanone	Fruity aroma	0.15 ± 0.07	0.19 ± 0.08	0.38 ± 0.02	0.02 ± 0.03	0.148
6-Methyl-2-heptanone	Cheese, musty	0.32 ± 0.08	0.49 ± 0.01	0.62 ± 0.02	0.66 ± 0.01	0.482
Cyclic hydrocarbons		7.48 ± 0.26	3.60 ± 0.38	2.76 ± 0.14	0.52 ± 0.02	0.112
4-Methylene-1,2-dimethylcyclopent-1-ene	Resinous fragrance	3.27 ± 0.11	1.19 ± 0.12	0.91 ± 0.05	0.50 ± 0.01	0.002
(2-Methylenecyclopropyl)methanol	Herbaceous and woody fragrance	4.21 ± 0.15	2.41 ± 0.26	1.85 ± 0.09	0.02 ± 0.01	0.043

Different letters indicate significant differences between groups after adding different levels of PMS to the feed (*p* < 0.05). Group K was fed with feed without PMS, Group S1 was fed with feed with 0.20% PMS, Group S2 was fed with feed with 0.25% PMS, and Group S3 was fed with feed with 0.30% PMS.

As demonstrated in Table 8, the application of the VIP >1 criterion facilitated the identification of nine compounds, namely: 1-hydrazinopropan-2-ol; 2-octanol; methyl 3-methylpentanoate; 2-ethylbutyl acetate; methyl heptanoate; 4-methylene-1,2-dimethylcyclopent-1-ene; (2-methylenecyclopropyl)methanol; 2-methylpentanoic acid and pentyl formate. In comparison to Group K, Group S1 exhibited increased contributions from 1-hydrazinopropan-2-ol; 2-octanol; methyl 3-methylpentanoate; 2-ethylbutyl acetate; and methyl heptanoate, Group S2 demonstrated enhanced contributions of the same compounds relative to Group K. Group S3, when compared to Group K, showed greater contributions from 1-hydrazinopropan-2-ol; 2-octanol; methyl 3-methylpentanoate; 2-ethylbutyl acetate; and 4-methylene-1,2-dimethylcyclopent-1-ene. Furthermore, Group S2, in comparison to Group S1, exhibited increased contributions from 1-hydrazinopropan-2-ol; 2-Octanol; and methyl 3-methylpentanoate; In comparison to the S3 group, the S1 group exhibited a higher contribution from 1-hydrazinopropan-2-ol, 2-octanol, methyl 3-methylpentanoate, and (2-methylenecyclopropyl)methanol.

**Table 8:** VIP values of the main volatile components of the longissimus dorsi muscle of Tibetan sheep by the addition of PMS.

Project	VIP					
	S1 vs. K	S2 vs. K	S3 vs. K	S2 vs. S1	S2 vs. S3	S1 vs. S3
1-Hydrazinopropan-2-ol	7.06	7.44	8.58	4.63	4.19	5.79
2-Octanol	5.85	5.99	4.61	5.57	6.18	5.61
Methyl 3-methylpentanoate	3.44	4.05	5.01	5.61	3.09	5.37
2-Ethylbutyl acetate	3.01	3.24	3.61	2.35	1.59	2.42
Methyl heptanoate	3.39	3.63	2.40	2.30	3.66	3.14
4-Methylene-1,2-dimethylcyclopent-1-ene	2.88	3.05	3.25	1.85	1.43	2.04
(2-Methylenecyclopropyl)methanol	2.84	2.52	3.40	2.65	2.30	3.16
2-Methylpentanoic acid	2.77	2.07	2.29	1.28	1.41	2.11
Pentyl formate	1.13	1.31	1.55	1.24	1.15	1.28

Group K was fed with feed without PMS, Group S1 was fed with feed with 0.20% PMS, Group S2 was fed with feed with 0.25% PMS, and Group S3 was fed with feed with 0.30% PMS.

### 3.8 Differential analysis of non-targeted metabolites in the longissimus dorsi muscle of Tibetan sheep following PMS supplementation

#### 3.8.1 Metabolic differential analysis

**Figure 5:**
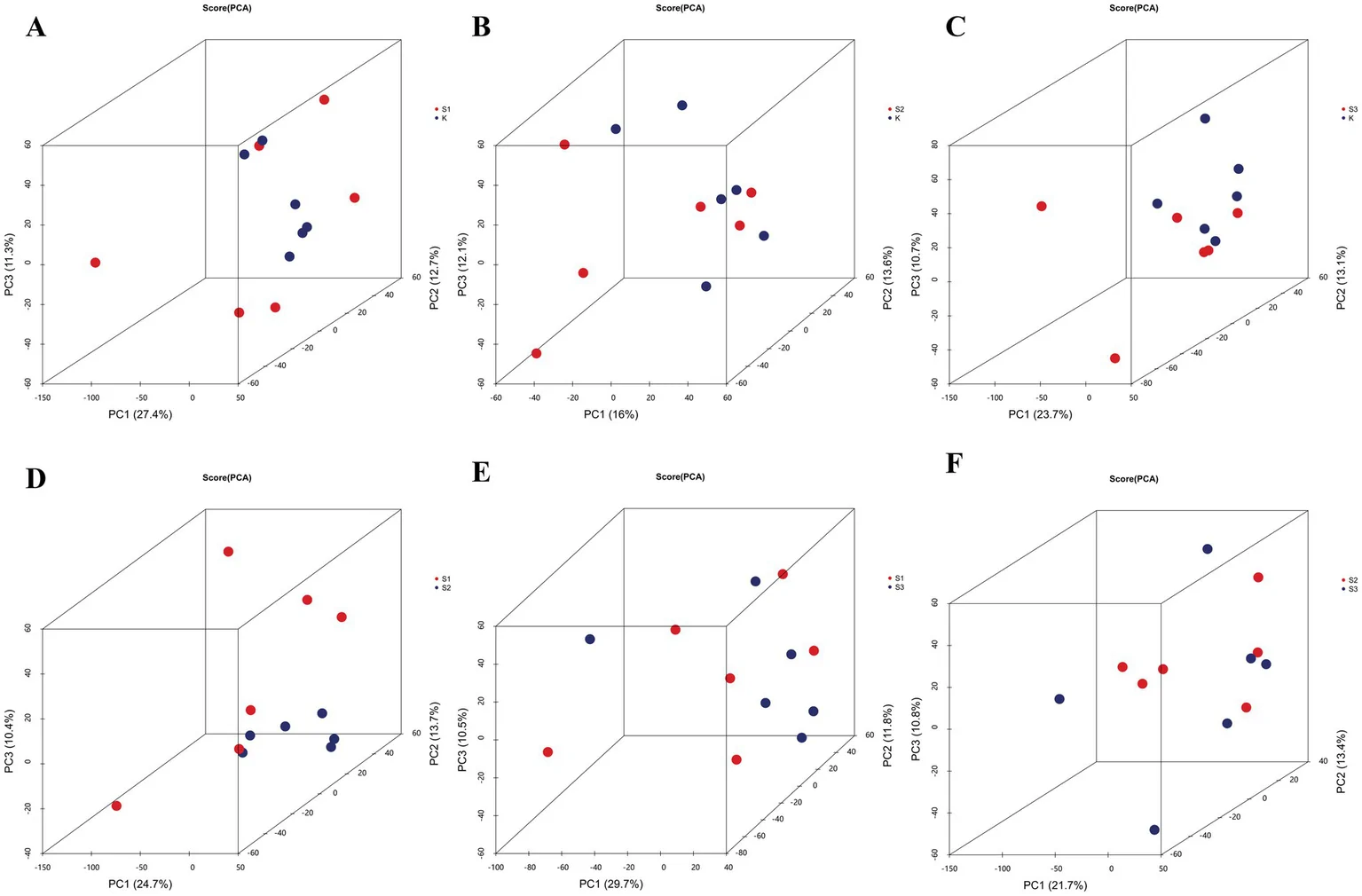
PCA score plot of untargeted metabolites in Tibetan lamb meat with different PMS added (**A**: K vs. S1; **B**: K vs. S2; **C**: K vs. S3; **D**: S1 vs. S2; **E**: S1 vs. S3; **F**: S2 vs. S3). 3D PCA score plots showing the distribution of two sample groups (represented by red circles and blue circles) across different pairwise comparisons. The first principal component (PC1), second principal component (PC2), and third principal component (PC3) are indicated on the axes, with the percentage of explained variance shown in parentheses for each component. Group K was fed with feed without PMS, Group S1 was fed with feed with 0.20% PMS, Group S2 was fed with feed with 0.25% PMS, and Group S3 was fed with feed with 0.30% PMS.

**Figure 6:**
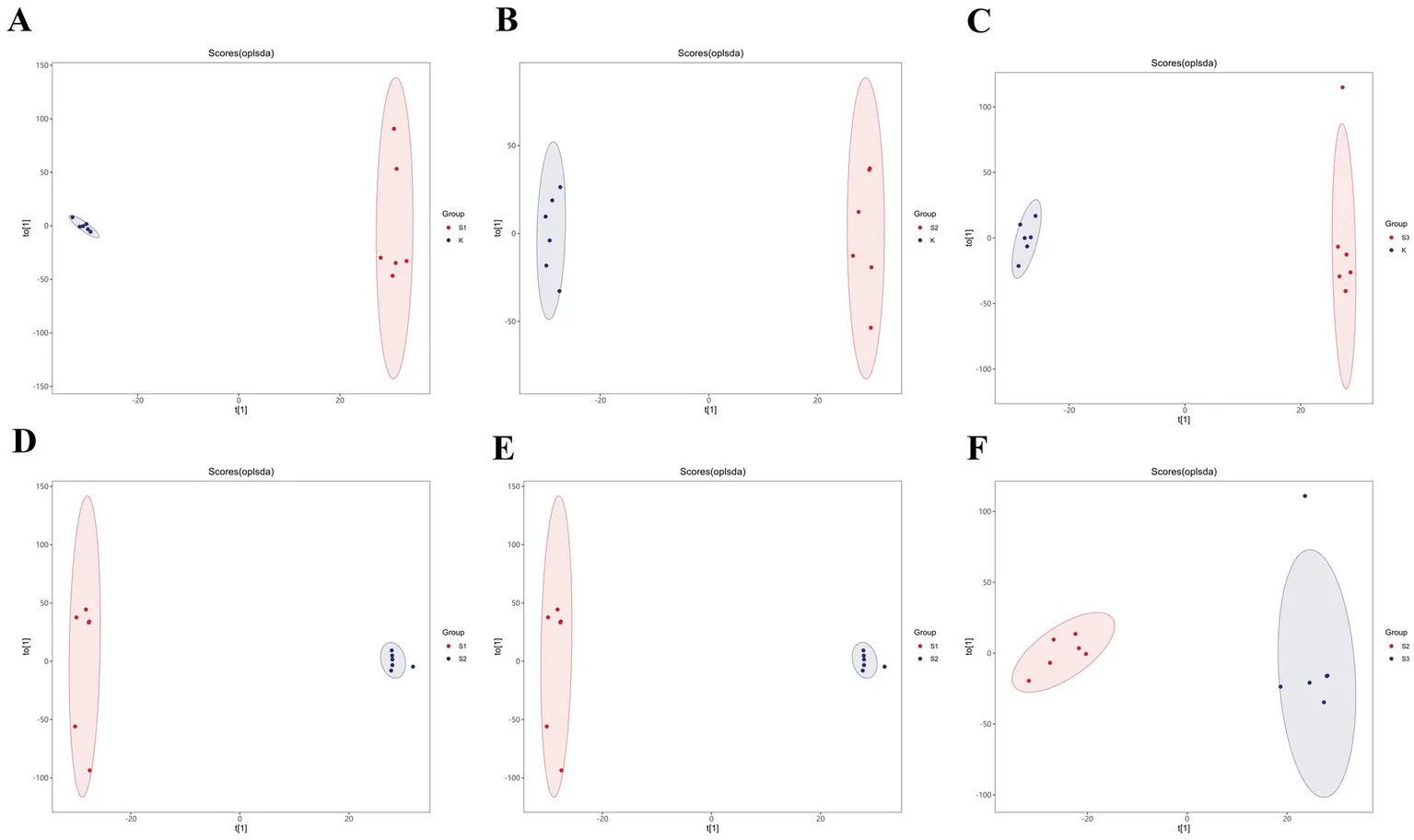
OPLS-DA plot of untargeted metabolites in Tibetan lamb meat with different PMS added (**A**: K and S1; **B**: K and S2; **C**: K and S3; **D**: S1 and S2; **E**: S1 and S3; **F**: S2 and S3). **(A–F)** OPLS-DA score plots showing the separation between two sample groups (red and blue dots) across different pairwise comparisons. The ellipses represent the 95% confidence intervals for each group. Group K was fed with feed without PMS, Group S1 was fed with feed with 0.20% PMS, Group S2 was fed with feed with 0.25% PMS, and Group S3 was fed with feed with 0.30% PMS.

To further investigate the effect of adding potassium magnesium sulfate (PMS) on the quality of the longest back muscle in Tibetan sheep, untargeted metabolomics analysis was performed using UHPLC-Orbitrap Exploris 480 mass spectrometer in both positive and negative ion modes. The experimental results showed that a total of 468 metabolites were identified in negative ion mode. To comprehensively evaluate the effects of different PMS addition levels on Tibetan sheep meat, subsequent analyses were conducted in negative ion mode. Unsupervised principal component analysis (PCA) was used to distinguish between experimental and control groups with different PMS addition levels, as shown in Figures 5A–F. The results showed significant differences among the four groups, indicating that the PMS-supplemented groups of Tibetan sheep had significantly different metabolic profile characteristics from the control group. In addition, to further explore the differences between groups, orthogonal partial least squares discriminant analysis (OPLS-DA) was used to evaluate metabolites in the meat samples, as shown in Figures 6A–F. Among them, t1 is the predicted principal component, mainly reflecting the metabolic variance related to grouping, and t2 is the orthogonal principal component. Each group of samples forms an independent cluster with clear boundaries. As shown in Supplementary Figure S2, *R*^2^*Y* represents the cumulative variance explained by the model, and the higher its value, the stronger the explanatory power of the model; *Q*^2^ represents the variance ratio of the data predicted by the current model. Usually, *Q*^2^ > 0.5 indicates that the model is stable and reliable, 0.3 < *Q*^2^ < 0.5 indicates moderate model stability, and *Q*^2^ < 0.3 indicates low model reliability. The results showed that each model exhibited significant explanatory power among the four groups, presenting clear intra group clustering and inter group dispersion characteristics.

#### 3.8.2 Metabolite quantity and chemical classification

**Figure 7:**
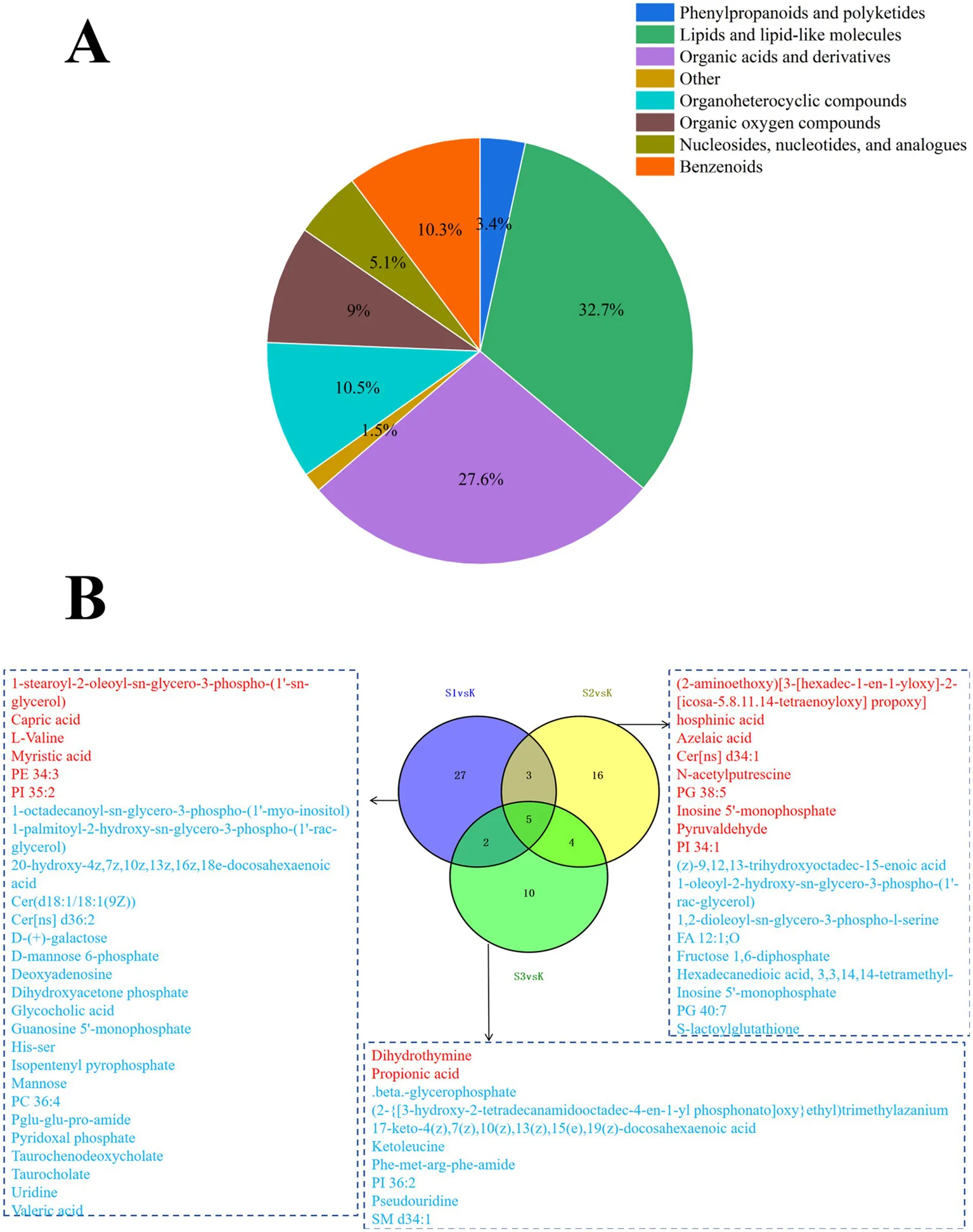
Proportion of identified compounds and Venn diagrams. **(A)** Pie chart showing the classification of all identified metabolites at the superclass level. **(B)** Venn diagram illustrating the number of differentially enriched metabolites in pairwise comparisons (S1 vs. K, S2 vs. K, S3 vs. K), along with a list of unique metabolites in each comparison. Red indicates upregulated lipid molecules, while blue represents downregulated lipid molecules. The K group was fed a diet without PMS, the S1 group was fed a diet containing 0.20% PMS, the S2 group was fed a diet containing 0.25% PMS, and the S3 group was fed a diet containing 0.30% PMS.

In the positive ion mode, a total of 468 metabolites were identified. As illustrated in Figure 7A, these metabolites were classified into eight principal categories: lipids and lipid-like molecules (32.7%), organic acids and derivatives (27.6%), organic heterocyclic compounds (10.5%), benzene and substituted derivatives (10.3%), oxygen-containing organic compounds (9.0%), nucleotides, nucleosides, and analogues (5.1%), phenylpropanoids and polyketides (3.4%), and others (1.5%). This distribution suggests that lipids and organic acids predominate across all four groups. To gain further insight into the metabolic alterations across the four sample groups and the overlap of significantly different metabolites identified in each comparative analysis, a Venn diagram is presented in Figure 7B. The comparisons of S1 vs. K, S2 vs. K, and S3 vs. K revealed five metabolites common to all three groups. Additionally, S1 and S2 shared three metabolites, S1 and S3 shared two, and S2 and S3 shared three. Furthermore, the comparisons S1 vs. K, S2 vs. K, and S3 vs. K identified 27 (6 up-regulated, 21 down-regulated), 16 (8 up-regulated, 9 down-regulated), and 10 (2 up-regulated, 8 down-regulated) unique differentially expressed metabolites, respectively.

#### 3.8.3 Identification and analysis of differentially expressed metabolites

Analysis of the differential metabolites across the four groups, as depicted in the volcano plots in Supplementary Figure S3, indicates distinct patterns of regulation. In comparison to Group K, Group S1 demonstrates an upregulation of organic oxides, alongside a downregulation of lipid molecules and their derivatives, as well as organic heterocyclic compounds (Supplementary Figure S3A). In contrast, Group S2 exhibits an upregulation of organic acids and their derivatives, while showing a downregulation of organic heterocyclic compounds, organic oxides, aromatic compounds, and lipid molecules and their derivatives (Supplementary Figure S3B). Group S3 is characterized by an upregulation of lipid molecules and their derivatives, as well as organic acids and their derivatives, whereas aromatic compounds, organic nitrogen compounds, organic oxides, phenylacetone, and polyketones are downregulated (Supplementary Figure S3C). When comparing S1 to S2, there is an observed upregulation of aromatic compounds and lipid molecules and their derivatives in S1, whereas organic heterocyclic compounds, organic acids and their derivatives, nucleotides, and nucleotide analogues are downregulated (Supplementary Figure S3D). In comparison to S3, S1 demonstrated an upregulation of organic acids and their derivatives, benzene compounds, and organic heterocyclic compounds, whereas nucleotides and nucleotide analogues were downregulated, as illustrated in Supplementary Figure S3E. Similarly, relative to S3, S2 exhibited an upregulation of benzene compounds, phenylpropanoids, and polyketones, while alkaloids and their derivatives, lipid molecules and derivatives, organic nitrogen compounds, and organic heterocyclic compounds were downregulated, as depicted in Supplementary Figure S3F.

#### 3.8.4 KEGG functional annotation and enrichment analysis of differentially expressed metabolites

**Figure 8:**
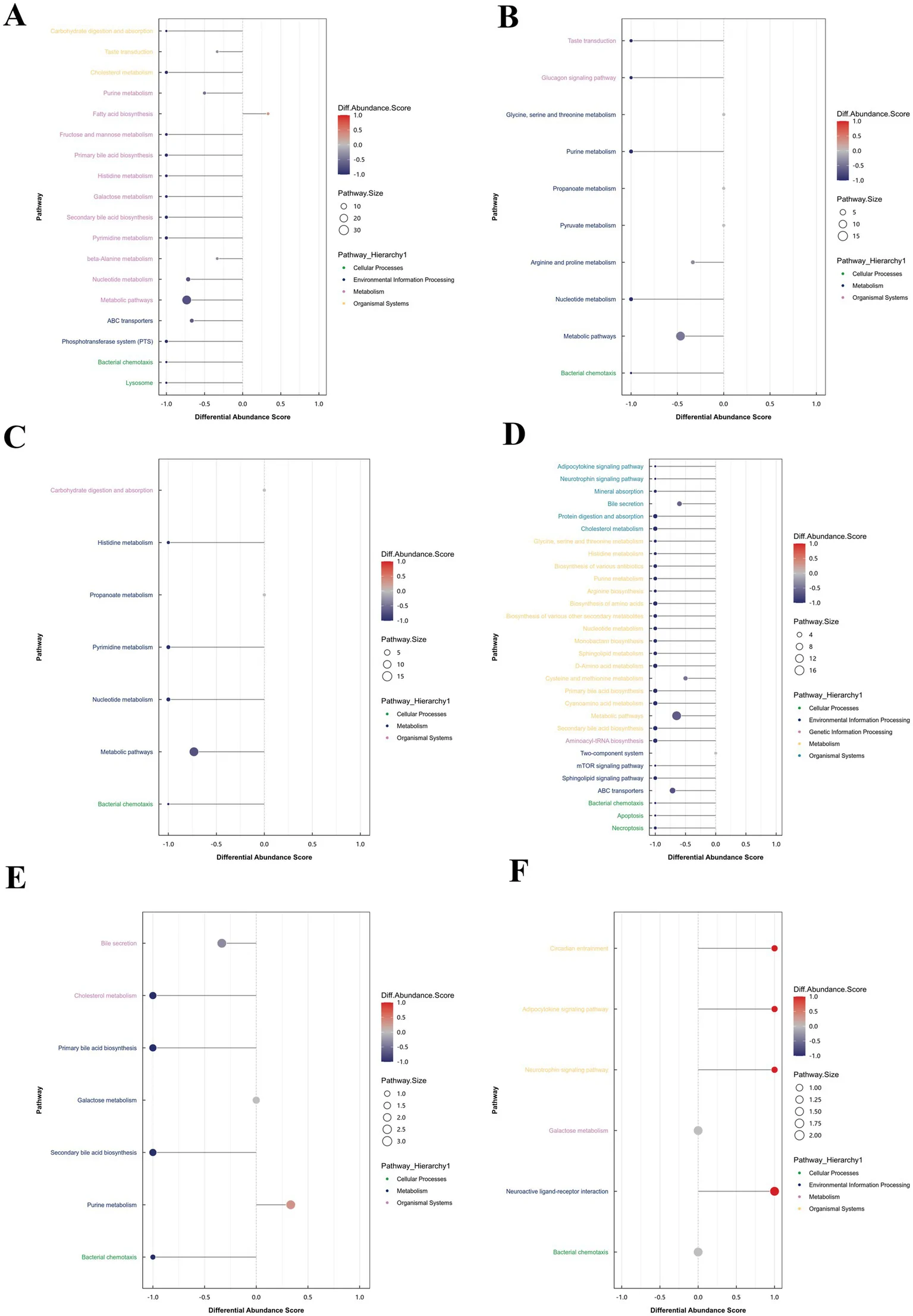
The differential abundance scores of four comparison groups (**A**: K vs. S1; **B**: K vs. S2; **C**: K vs. S3; **D**: SI vs. S2; **E**: S1 vs. S3; **F**: S2 vs. S3). **(A–F)** Bar plots showing the differential abundance scores of key metabolic pathways in different pairwise comparisons. Group K was fed with feed without PMS, Group S1 was fed with feed with 0.20% PMS, Group S2 was fed with feed with 0.25% PMS, and Group S3 was fed with feed with 0.30% PMS.

The KEGG pathway enrichment analysis presented in Figure 8 identified enriched pathways in Tibetan sheep subjected to four distinct levels of PMS. Relative to the K group, the S1 group demonstrated significant enrichment in pathways associated with fatty acid biosynthesis, bile acid biosynthesis, nucleotide metabolism, carbohydrate metabolism, cholesterol metabolism, phosphotransferase systems, and ABC transporters (Figure 8A). In contrast, the S2 group predominantly exhibited enrichment in pathways related to purine metabolism, nucleotide metabolism, and arginine and proline metabolism (Figure 8B). The S3 group primarily showed enrichment in pathways associated with pyrimidine metabolism and nucleotide metabolism (Figure 8C). When compared to S2, the S1 group displayed notable enrichment in pathways related to sphingolipid metabolism, bile acid biosynthesis, purine metabolism, protein digestion and absorption, nucleotide metabolism, cysteine and methionine metabolism, D-amino acid metabolism, arginine metabolism, cholesterol metabolism, antibiotic biosynthesis, amino acid biosynthesis, ABC transporters, aminoacyl-tRNA synthesis, and various other secondary metabolite biosyntheses (Figure 8D); In comparison to S3, the majority of genes in S1 are linked to purine metabolism, bile secretion, and cholesterol metabolism (refer to Figure 8E). Similarly, when compared to S3, the majority of genes in S2 are associated with secondary metabolite biosynthesis, amino acid metabolism, and adipokine signaling pathways (refer to Figure 8F).

To further elucidate the impact of PMS supplementation on metabolic pathways in Tibetan sheep, differential abundance scores (DA scores) were employed to analyze the four experimental groups. As presented in Table 9, relative to the K group, 11 metabolic pathways were significantly downregulated in S1 (DA score >0.5, *p* < 0.05). These pathways included nucleotide metabolism, cholesterol metabolism, the phosphotransferase system (PTS), β-alanine metabolism, taste transmission, ABC transport, pyrimidine metabolism, secondary bile acid biosynthesis, primary bile acid biosynthesis, purine metabolism, galactose metabolism, and fructose and mannose metabolism. The downregulated metabolites within these pathways comprised guanine nucleotides and glycocholic acid. Conversely, in S1, one metabolic pathway was upregulated: bile acid biosynthesis. The metabolites that were upregulated included oleic acid, palmitic acid, and myristic acid. In the S2 group, five metabolic pathways were observed to be downregulated, namely purine metabolism, nucleotide metabolism, arginine and proline metabolism, and taste transmission. The downregulated metabolites included inosine monophosphate, xanthine, adenosine ribosyl diphosphate, and creatinine. In contrast, the S3 group exhibited downregulation in four metabolic pathways, specifically histidine metabolism, pyrimidine metabolism, and nucleotide metabolism, with the associated metabolites being xanthine, pseudouridine, and L-aspartic acid.

**Table 9:** Compares the changes of differential metabolites in the main metabolic pathways in the four groups of samples (the absolute differential abundance score of all metabolic pathways is ≥0.5).

Metabolic pathway	Metabolites
Up-regulation of S1 group	S1 vs. K
Fatty acid biosynthesis	Capric acid, Cis-9-palmitoleic acid, myristic acid
Down-regulation of S1 group	S1 vs. K
Secondary bile acid biosynthesis	Glycocholic acid, taurochenodeoxycholate taurocholate, glycocholate
Pyrimidine metabolism	Cytidine 5′-monophosphate, pseudouridine, uridine
Purine metabolism	Deoxyadenosine, guanosine 5′-monophosphate 2′-deoxyinosine, adenine
Primary bile acid biosynthesis	Glycocholic acid, taurochenodeoxycholate, taurocholate glycocholate
Phosphotransferase system (PTS)	D-(+)-galactose, D-mannose 6-phosphate, mannose, maltose
Nucleotide metabolism	Cytidine 5′-monophosphate, deoxyadenosine, adenine Guanosine 5′-monophosphate, uridine, 2′-deoxyinosine
Galactose metabolism	D-(+)-galactose, dihydroxyacetone phosphate mannose
Fructose and mannose metabolism	D-mannose 6-phosphate Dihydroxyacetone phosphate, mannose
Cholesterol metabolism	Glycocholic acid, taurochenodeoxycholate, taurocholate, glycocholate
Beta-alanine metabolism	Gamma-aminobutyric acid
ABC transporters	L-Valine, mannose, uridine 2′-deoxyinosine, maltose deoxyadenosine
Down-regulation of S2 group	S2 vs. K
Purine metabolism	Inosine 5′-monophosphate, xanthine, ADP-ribose
Nucleotide metabolism	Cytidine 5′-monophosphate Inosine 5′-monophosphate, xanthine
Arginine and proline metabolism	Guanidoacetic acid, N-acetylputrescine, creatinine
Taste transduction	Guanosine 5′-monophosphate, gamma-aminobutyric acid maltose
Bacterial chemotaxis	Malt sugar Alpha-D-glucopyranosyl-(1 → 4)-D-glucopyranose
Down-regulation of S2 group	S3 vs. K
Pyrimidine metabolism	Cytidine 5′-monophosphate, pseudouridine 2′-deoxycytidine
Nucleotide metabolism	Cytidine 5′-monophosphate, xanthine, 2′-deoxycytidine
Bacterial chemotaxis	Malt sugar, alpha-D-glucopyranosyl-(1 → 4)-D-glucopyranose
Histidine metabolism	L-Aspartic acid
Down-regulation of S1 group	S1 vs. S2
Sphingolipid signaling pathway	DL-serine, sphingosine, palmitic acid
Sphingolipid metabolism	DL-serine, sphingosine, palmitic acid
Secondary bile acid biosynthesis	Glycochenodeoxycholate, glycocholic acid, taurocholate Taurochenodeoxycholate
Purine metabolism	Inosine, hypoxanthine
Protein digestion and absorption	DL-serine, L-aspartic acid, arginine, phenylalanine
Primary bile acid biosynthesis	Glycochenodeoxycholate, glycocholic acid, taurocholate Taurochenodeoxycholate
Nucleotide metabolism	Inosine, uridine, hypoxanthine
Monobactam biosynthesis	DL-serine, L-aspartic acid, arginine
D-Amino acid metabolism	DL-serine, L-aspartic acid, arginine, phenylalanine
Cysteine and methionine metabolism	DL-serine, L-aspartic acid, L-glutathione, reduced S-methyl-5′-thioadenosine
Cyanoamino acid metabolism	DL-serine, L-aspartic acid, mandelonitrile, phenylalanine
Cholesterol metabolism	Glycochenodeoxycholate, glycocholic acid taurochenodeoxycholate, taurocholate
Biosynthesis of various other secondary metabolites	DL-serine, L-aspartic acid, phenylalanine
Biosynthesis of various antibiotics	DL-serine, L-aspartic acid, arginine
Biosynthesis of amino acids	DL-serine, L-aspartic acid, arginine, phenylalanine
Bile secretion	Glycochenodeoxycholate, glycocholic acid, taurocholate Taurochenodeoxycholate, L-glutathione, reduced
Aminoacyl-tRNA biosynthesis	DL-serine, L-aspartic acid, arginine, phenylalanine
ABC transporters	DL-serine, inosine, L-aspartic acid, uridine, arginine L-glutathione, reduced, phenylalanine
Down-regulation of S2 group	S1 vs. S3
Secondary bile acid biosynthesis	Glycochenodeoxycholate, glycocholic acid, taurocholate Taurochenodeoxycholate
Primary bile acid biosynthesis	Glycochenodeoxycholate, glycocholic acid, taurocholate Taurochenodeoxycholate
Cholesterol metabolism	Glycochenodeoxycholate, glycocholic acid, taurocholate taurochenodeoxycholate
Up-regulation of S1 group	S2 vs. S3
Neuroactive ligand-receptor interaction	L-Aspartate, M-L-aspartic acid, N-2-aminosuccinic acid L-AspMelatonin, N-acetyl-5-methoxytryptamine
Neurotrophin signaling pathway	Acylsphingosine, ceramide, N-acylsphing-4-enine (4E)-sphing-4-enine ceramide
Circadian entrainment	N-Acetyl-5-methoxytryptamine
Adipocytokine signaling pathway	Acylsphingosine, ceramide, N-acylsphing-4-enine (4E)-sphing-4-enine ceramide

Group K was fed with feed without PMS, Group S1 was fed with feed with 0.20% PMS, Group S2 was fed with feed with 0.25% PMS, and Group S3 was fed with feed with 0.30% PMS.

Relative to S2, the S1 group demonstrated downregulation in 18 metabolic pathways, including sphingolipid metabolism, bile acid metabolism, purine metabolism, cysteine and methionine metabolism, nucleotide metabolism, cholesterol metabolism, and antibiotic biosynthesis. The metabolites involved in these pathways were arginine, phenylalanine, glycocholic acid, taurocholate, DL-serine, and L-aspartic acid. When compared to S1, the S3 group showed downregulation in three metabolic pathways, specifically bile acid metabolism and cholesterol metabolism, with the metabolites being glycocholic acid and taurocholate. In comparison to Group S3, four metabolic pathways were upregulated in Group S2, notably the neurotrophic signaling pathway and the adipocyte signaling factor pathway. The metabolites involved included N-acylsphingosine, N-sphingomyelin, N-acylsphingosine-4-enoyl-L-carnitine, and (4E)-sphingosine-4-enoyl-L-carnitine.

Overall, Groups S1 and S2 exhibited a primary focus on the biosynthesis of fatty acids, adipokine signaling pathways, and the upregulation of sphingomyelin metabolism. In contrast, pathways related to purine metabolism, nucleotide metabolism, and the metabolism of arginine and proline were downregulated. Group S3 did not exhibit any distinct core pathways, as evidenced by a broad metabolic suppression. These findings suggest that varying concentrations of PMS in the diet influence fatty acid biosynthesis, adipokine signaling pathways, and sphingomyelin metabolism in the longissimus dorsi muscle of Tibetan sheep. This further substantiates the significant impact of PMS supplementation on muscle metabolism in Tibetan sheep.

### 3.9 Correlation analysis

**Figure 9:**
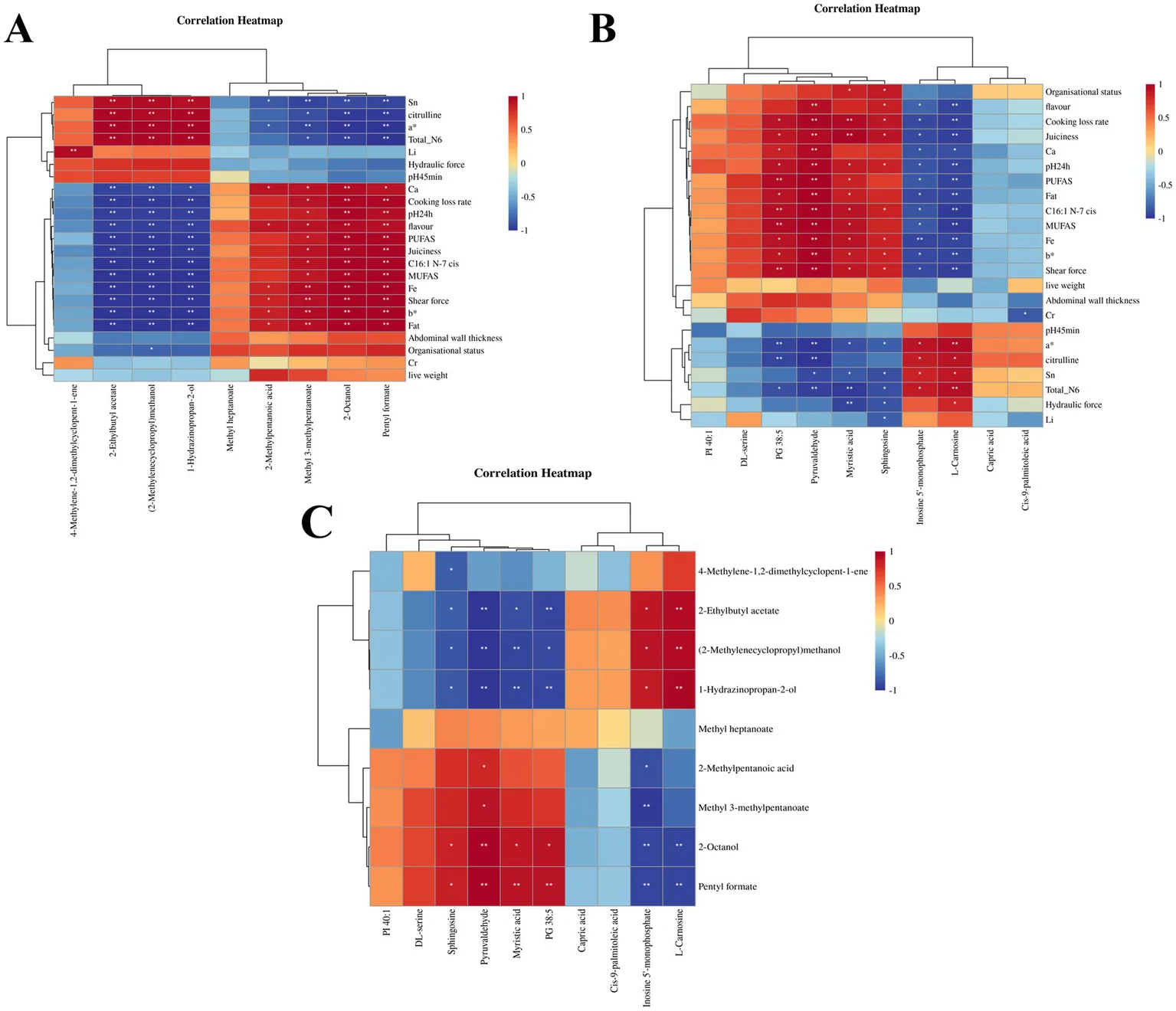
Correlation clustering heatmap between muscle metabolites and meat quality. **(A)** Meat quality and volatile substances. **(B)** Meat quality and non-volatile substances. **(C)** The correlation between volatile and non-volatile substances. Red and blue represent positive and negative correlations, respectively. The color intensity is directly proportional to the correlation value, with ^*^p < 0.05 and ^**^p < 0.01. Group K was fed with feed without PMS, Group S1 was fed with feed containing 0.20% PMS, Group S2 was fed with feed containing 0.25% PMS, and Group S3 was fed with feed containing 0.30% PMS.

To examine the relationships between PMS supplementation and the quality of muscle meat in Tibetan sheep, including both volatile and non-volatile metabolites and their interactions, correlation analyses were performed, as illustrated in Figure 9. Figure 9A presents a correlation clustering heatmap that illustrates the associations between volatile flavor compounds and meat quality parameters in Tibetan sheep muscle. Notably, 2-ethylbutyl acetate, 1-hydrazinopropan-2-ol, and (2-methylenecyclopropyl)methanol exhibited significant positive correlations with the mineral element Sn, color parameter a^*^, and n-6 polyunsaturated fatty acids (PUFAs). Conversely, these compounds were significantly negatively correlated with the mineral elements Fe and Ca, as well as with juiciness, total PUFAs, C16:1 n-7 cis, monounsaturated fatty acids (MUFAs), shear force, color parameter b^*^, fat content, pH at 24 h (Ph24h), and cooking loss rate. Additionally, 4-methylene-1,2-dimethylcyclopent-1-ene, demonstrated significant positive correlations with the mineral element Li and the amino acid citrulline. The compounds methyl 3-methylpentanoate, 2-methylpentanoic acid, 2-octanol, and pentyl formate demonstrated significant negative correlations with the mineral element tin (Sn), the color parameter a^*^, and n-6 polyunsaturated fatty acids (n-6 PUFA). Conversely, these compounds exhibited significant positive correlations with the mineral elements iron (Fe) and calcium (Ca), as well as with citrulline, polyunsaturated fatty acids (PUFAs), C16:1 n-7 cis, monounsaturated fatty acids (MUFAs), shear force, the color parameter b^*^, fat content, pH at 24 h (Ph24h), and cooking loss rate.

Figure 9B depicts the relationships between non-volatile metabolites in Tibetan sheep muscle and meat quality. It was observed that PG38:5 and pyruvaldehyde showed significant positive correlations with cooking loss rate, juiciness, calcium (Ca), pH at 24 h (pH24h), PUFAs, fat content, C16:1 n-7 cis, MUFAs, shear force, the color parameter b^*^, and the mineral element iron (Fe) in Tibetan sheep muscle. These compounds also exhibited significant negative correlations with the color parameter a^*^, citrulline, and total n-6. Additionally, myristic acid and sphingosine demonstrated significant positive correlations with organizational status, cooking loss rate, juiciness, pH at 24 h (pH24h), C16:1 n-7 cis, shear force, the color parameter b^*^, and the mineral element iron (Fe). They also showed significant negative correlations with hydraulic force, the color parameter a^*^, total n-6, and the mineral element tin (Sn). Inosine 5′-monophosphate and L-carnosine demonstrated significant negative correlations with cooking loss rate, juiciness, calcium (Ca), pH at 24 h (pH24h), polyunsaturated fatty acids (PUFAs), fat content, palmitoleic acid (C16:1 n-7 cis), monounsaturated fatty acids (MUFAs), shear force, color parameter b^*^, and the mineral element iron (Fe). Conversely, they exhibited significant positive correlations with color parameter a^*^, citrulline, total n-6 fatty acids (Total_N6), and the mineral element (Sn).

As depicted in Figure 9C, myristic acid, sphingosine, phosphatidylglycerol (PG 38:5), and pyruvaldehyde displayed significant negative correlations with 2-ethylbutyl acetate, 1-hydrazinopropan-2-ol, and (2-methylenecyclopropyl)methanol. In contrast, they showed significant positive correlations with pentyl formate, and 2-octanol. Additionally, inosine 5′-monophosphate and L-carnosine exhibited significant positive correlations with 2-ethylbutyl acetate, 1-hydrazinopropan-2-ol and (2-methylenecyclopropyl)methanol, while demonstrating significant negative correlations with pentyl formate, and 2-octanol.

## 4 Discussion

This study conducted a systematic investigation into the effects of incorporating PMS into the diets of Tibetan sheep, focusing on carcass quality, meat quality, nutritional quality, and both volatile and non-volatile metabolites. Suggest that PMS was associated with enhanced carcass quality, as evidenced by increased abdominal wall thickness and backfat thickness, as well as improvements in edible quality parameters such as pH, color, tenderness, and water-holding capacity. Additionally, the nutritional quality, particularly fat content, was improved. The study also observed an optimization in the composition of volatile and non-volatile metabolites, which contributed to an enhanced flavor profile of Tibetan sheep meat.

In terms of carcass quality, abdominal wall thickness and backfat thickness are critical indicators of fat deposition characteristics in ruminant carcasses (22). Abdominal wall thickness refers to the combined thickness of subcutaneous fat and muscle layers in the animal’s abdomen, serving as a vital indicator of energy metabolism status and fat deposition patterns in ruminants. Backfat thickness, defined as the thickness of subcutaneous fat on the back of Tibetan sheep, is an essential metric for evaluating body fat deposition and overall carcass fatness. Research has demonstrated a correlation between increased abdominal wall thickness and diets high in energy or protein, which facilitate the accumulation of fat and muscle (23). The present study observed that treatment with potassium magnesium sulfate led to a 13.13% increase in abdominal wall thickness in Tibetan sheep compared to the control group, with a notable upward trend in backfat thickness in the medium-to-high dose groups. Backfat thickness was significantly increased by 7.41% relative to the control group. Non-targeted metabolomics analysis revealed a significant upregulation of fatty acid biosynthesis in the S1 group, with metabolites such as myristic acid, palmitic acid, and decanoic acid significantly elevated, thereby enhancing the fatty acid biosynthesis pathway. These findings are consistent with previous research on the role of minerals in regulating fat deposition (23). Furthermore, Du et al. (24) reported that the inclusion of 1% L-citrulline in the diet of finishing pigs resulted in a significant increase in both carcass weight and average backfat thickness. Increasing the dietary electrolyte balance (dEB) in wheat-based diets has been shown to linearly increase backfat thickness in pigs, a phenomenon associated with the regulation of insulin secretion and the promotion of lipogenesis through Na^+^/K^+^ mechanisms (25). This observation aligns with our findings that PMS supplementation concurrently enhanced carcass traits and metabolic profiles. Metabolically, palmitic acid (C16:0) and stearic acid (C18:0) are the primary end products of *de novo* fatty acid synthesis in animals, and their increased levels may be associated with heightened FAS activity (26).

In terms of growth performance, dietary supplementation with PMS significantly influenced feed intake and feed efficiency. The average daily feed intake (ADFI) in all PMS-supplemented groups was significantly higher than that in the control group (*p* < 0.05), with the S2 group showing the highest ADFI. Regarding feed conversion ratio (FCR), the S1 group (0.20% PMS) exhibited a significantly lower FCR compared to the control group (*p* < 0.05), indicating improved feed efficiency. Consistent with our findings, studies have shown that supplementation with an alkaline mineral complex (3 g/d per lamb) significantly increased average daily gain (ADG) and decreased FCR in fattening lambs, while also improving meat redness (a^*^) and intramuscular fat content (27). Although ADG showed an increasing trend in PMS-supplemented groups, the differences were not statistically significant. This suggests that the primary effect of PMS on growth performance was mediated through increased feed intake and improved feed conversion efficiency, rather than directly promoting growth. Studies have also shown that the concentrations of macro-minerals (particularly K and P) in winter forage are below recommended levels, and mineral supplementation may help balance these deficiencies and improve production performance (28). The improved FCR in the S1 group is also consistent with the upregulation of fatty acid biosynthesis pathways observed in the metabolomics analysis, suggesting that enhanced lipid metabolism may contribute to more efficient energy utilization.

Meat quality is a critical parameter in evaluating meat superiority. Our study demonstrated that PMS supplementation in the diet of Tibetan sheep resulted in alterations in meat quality, as evidenced by changes in pH, a^*^, b^*^, water-holding capacity, and shear force. Previous studies have indicated that lower pH levels, tougher and drier meat, reduced shelf life, and inferior meat appearance, thereby impacting meat quality and safety (29). Post-slaughter, the cessation of oxygen supply shifts cellular energy metabolism toward glycolysis, leading to glycogen breakdown and lactic acid production, which in turn causes a decline in pH. When pH changes stabilize, it signifies the complete depletion of glycolytic activity (30). Experimental data indicated a decreasing trend in pH across all four groups at 45 min post-slaughter. However, after 24 h of aging, Group S2 exhibited the highest pH, suggesting a moderate rate of glycolysis and a higher ultimate pH. This observation is consistent with the findings of Ramos Z, who reported a positive correlation between meat buffering capacity and final pH (31). Concurrently, metabolomic analyses revealed regulated levels of acetaldehyde, a precursor of lactic acid, along with the accumulation of the buffering agent L-myo-inositol in the S2 group. Correlation analysis demonstrated significant positive associations between L-myo-inositol and pH at 24 h, as well as between acetaldehyde, a glycolytic byproduct, and pH at 24 h, collectively elucidating the metabolic basis for the superior post-slaughter pH observed in the S2 group.

Meat color is a primary criterion for consumers in evaluating meat quality, with visually appealing meat more effectively stimulating purchase intent. Generally, higher a^*^ values and lower b^*^ and L^*^ values are preferred for meat (32). In this experiment, while L^*^ values did not exhibit significant differences among the four groups, the S2 group demonstrated higher a^*^ values compared to the K group, and lower b^*^ values than both the K and S3 groups. This phenomenon is hypothesized to result from elevated pH levels inhibiting mitochondrial oxygenation, thereby reducing MetMb accumulation, which significantly increases a^*^ (redness) and decreases b^*^ (yellowness). These findings are consistent with those reported by Joseph et al. (33). Concerning the water-holding capacity of meat, the water-binding capacity displayed a gradual upward trend. It is postulated that this may be attributed to the addition of PMS, which enhances the osmotic environment within muscle cells, thereby facilitating improved water retention and promoting the synthesis of certain proteins related to water-binding in muscle tissue, ultimately enhancing water-binding capacity. This hypothesis is supported by the significant upregulation of membrane phospholipids, such as phosphatidylinositol (PI 34:1) and phosphatidylglycerol (PG 38:5), in metabolites, which are involved in membrane structure and osmotic regulation, collectively providing a foundation for cellular water retention. Moreover, the tenderness of lamb is a critical determinant of consumer acceptance and satisfaction, with shear force serving as a primary indicator of meat tenderness (34). Several factors influence meat tenderness, including muscle fiber structure, protein degradation, fat content, and pH fluctuations (35). Experimental findings revealed that the shear force in the S2 group was lower than that in the K group. Research suggests a positive correlation between muscle fiber cross-sectional area (CSA) and shear force, especially during the initial post-slaughter maturation phase. Additionally, the degradation of structural proteins is pivotal in enhancing tenderness during post-slaughter maturation. Studies have demonstrated a positive correlation between intramuscular fat (IMF) content and sensory tenderness, with each 1% increase in IMF resulting in a 2–3 point enhancement in tenderness scores (36). Concurrently, the rate of pH change significantly affects meat tenderness, with a rapid post-slaughter pH decline typically leading to increased shear force. Moreover, pH demonstrates significant correlations with collagen solubility and intramuscular fat (IMF) content, indicating that these factors collectively influence meat tenderness (37).

The fat content within muscle tissue plays a critical role in determining meat quality, as it is closely associated with attributes such as tenderness and juiciness. In this study, dietary supplementation with PMS resulted in a significant increase in fat content within the longissimus dorsi muscle of Tibetan sheep, accompanied by concurrent increases in backfat thickness (+7.41%) and abdominal wall thickness (+13.13%). Non-targeted metabolomics KEGG analysis further identified a significant upregulation of the adipokine signaling pathway in the S2 group. Upregulation of this pathway was associated with enhanced insulin sensitivity and anabolic metabolism, thereby systematically creating an optimal metabolic environment for intramuscular fat deposition. Simultaneously, there was a significant downregulation of purine metabolism, nucleotide metabolism, and the metabolism of arginine and proline. This downregulation is hypothesized to decrease energy-intensive cell proliferation, thereby redirecting conserved resources toward fat synthesis. Correlation analysis demonstrated positive associations between myristic acid, a compound linked to fat synthesis, and fat content. Conversely, negative correlations were identified for the umami compound inosine monophosphate (IMP) and the buffering agent L-mycoprolin. Based on these findings, we propose that dietary supplementation with PMS may enhance fat synthesis, thereby increasing overall carcass fat deposition, improving acid–base balance, and facilitating the accumulation of flavor precursors. Previous research has shown that diets enriched with lactic acid bacteria significantly elevate intramuscular fat (IMF) content in Sunit sheep and optimize fatty acid composition by modulating lipid metabolic pathways (38). Furthermore, betaine has been found to promote IMF deposition in pigs by upregulating the expression of key lipogenic enzymes, such as fatty acid synthase (FAS) and acetyl-CoA carboxylase (ACC) (39). Consequently, we hypothesize that dietary PMS supplementation may concurrently enhance intramuscular fat deposition in Tibetan sheep muscle and carcass fat by activating the expression of critical lipogenic enzymes and regulating lipid metabolic pathways, ultimately improving meat tenderness and juiciness.

The sensory quality of lamb meat, a critical parameter for assessing overall meat quality, is evaluated through various indicators, including flavor, color, texture, juiciness, and overall acceptability. Experimental findings demonstrate that the sensory quality of Group S2 surpassed that of the other three groups. Non-targeted metabolomics analysis revealed a significant increase in inosine monophosphate (IMP), a compound associated with umami flavor, within the S2 group. This suggests that dietary supplementation with PMS substantially elevated intramuscular fat (IMF), thereby enhancing the umami flavor profile of Tibetan sheep. This observation is consistent with the study by Wang et al. (40), which reported that feed additives significantly increased IMF content in broiler chickens, resulting in improved umami flavor ratings. This enhancement was strongly correlated with indicators of edible quality. Notably, the intramuscular fat content in the S2 group was significantly higher than that of the control group (*p* < 0.05). This increase may be attributed to the release of fat-soluble aldehydes, ketones, and other flavor compounds during mastication, which not only lubricates muscle fibers but also intensifies flavor and juiciness (41). This study corroborates the findings of Priolo et al. (42), who reported that a 0.5% increase in intramuscular fat (IMF) is associated with a 0.6–0.8 point enhancement in juiciness sensory scores. This improvement is attributed to the release of ketones during mastication, which lubricates muscle fibers and subsequently enhances flavor intensity and juiciness (41). Furthermore, Group S2 demonstrated elevated a^*^ values and reduced b^*^ values, indicative of a bright red coloration. This observation is consistent with the results of Ponnampalam et al. (43), who noted that optimal meat color scores occur when a^*^ exceeds 14.5. Additionally, the pH measured at 24 h post-mortem was significantly higher in the experimental group (6.18) compared to the control group (5.75), suggesting a potential reduction in protein denaturation, preservation of muscle fiber integrity, and a resultant finer tissue texture.

The inclusion of mineral elements in the diet has a substantial impact on the growth, health, and production performance of Tibetan sheep. In this study, PMS supplementation led to increased levels of elements such as iron (Fe) and calcium (Ca). Moreover, research indicates that dietary potassium supplementation significantly influences calcium absorption capacity. In experimental studies involving sheep, the potassium content in the diet of the potassium supplementation group was 3.1%, compared to 1.4% in the control group (44). At first, this appears contradictory to our observation of increased muscle Ca content in PMS-supplemented groups. However, this discrepancy may be explained by tissue-specific regulation of mineral homeostasis. While high potassium intake may reduce intestinal calcium absorption at the systemic level (45), the increase in muscle calcium content likely reflects enhanced muscle metabolic activity and upregulation of calcium-binding proteins in response to improved growth performance and muscle development. Furthermore, potassium plays a key role in maintaining cellular osmolarity and activating metabolic enzymes, which may indirectly promote calcium retention in muscle tissue (46). Thus, the effects of PMS on calcium metabolism appear to be tissue-dependent, with systemic and local regulatory mechanisms operating simultaneously. Potassium intake may also indirectly influence iron absorption, as iron is a vital component of hemoglobin and myoglobin, essential for oxygen transport and storage. Excessive potassium consumption may enhance renal excretion of minerals, thereby impacting iron absorption and utilization. Consequently, we hypothesize that dietary potassium magnesium sulfate (PMS) supplementation may indirectly modulate the absorption and metabolic balance of key minerals such as iron and calcium in Tibetan sheep, ultimately affecting their growth performance and meat quality.

Amino acids are crucial constituents that enhance the flavor and nutritional value of lamb meat (47). This study observed that alanine levels in Group S2 were significantly higher than in the other three groups, while citrulline levels in Group K were significantly elevated compared to Groups S2 and S3. Alanine is primarily involved in gluconeogenesis and nitrogen metabolism, while citrulline functions as a vital intermediate in the urea cycle and nitric oxide (NO) synthesis (48). Studies have demonstrated that in the liver and kidneys, citrulline can be converted into arginine through the urea cycle. Subsequently, arginine can be further degraded into urea and ornithine, with ornithine being transaminated to form alanine (49). Magnesium acts as a cofactor for numerous enzymes, whereas sulfur is an integral component of sulfur-containing amino acids. It is postulated that magnesium might influence the conversion of citrulline by modulating the activity of enzymes involved in the urea cycle, while sulfur could indirectly affect alanine levels by impacting the metabolism of sulfur-containing amino acids (50). It should be noted that this proposed mechanism is hypothetical and requires direct experimental validation. Ongoing follow-up studies in our laboratory are currently measuring the activities of FAS, ACC, and HSL using commercial ELISA kits to directly test this hypothesis.

Fatty acids, essential nutritional constituents of meat products, are classified into saturated fatty acids, monounsaturated fatty acids, and polyunsaturated fatty acids. Research has indicated that the levels of saturated fatty acids in pork tenderloin increase with elevated PKM levels in the diet (51). This study yielded results consistent with those observed in Tibetan sheep meat, where supplementation with PMS led to a significant increase in saturated fatty acids, including palmitic acid, stearic acid, and myristic acid. This outcome is associated with the upregulation of fatty acid biosynthesis pathways as identified through metabolomic analysis, suggesting a potential connection to the activation of fatty acid synthase (FAS) by magnesium ions. Magnesium, serving as a cofactor for over 300 enzymes, plays a critical role in the regulation of fatty acid synthesis. Evidence indicates that magnesium ions enhance FAS activity, facilitating the elongation of acetyl-CoA into long-chain saturated fatty acids (52). Concurrently, magnesium contributes to the stabilization of cell membrane fluidity, thereby reducing β-oxidation and resulting in the accumulation of C16:0 and C18:0 within tissues. Moreover, sulfate ions are implicated in the indirect regulation of adipocyte differentiation-related gene expression (PPARγ, C/EBPα) through their involvement in sulfur-containing amino acid metabolism (53), which promotes the differentiation of preadipocytes into mature adipocytes and consequently enhances the synthesis of C14:0. Simultaneously, research has demonstrated that the S2 group exhibited significantly elevated levels of unsaturated fatty acids, DPA, and DHA in comparison to the K group. The content of polyunsaturated fatty acids is a critical indicator for assessing the nutritional value of lamb meat and plays an essential role in mitigating cardiovascular and cerebrovascular diseases (54). Evidence suggests that DHA and DPA contribute to cardiovascular health through various mechanisms, including lipid-lowering effects, anti-inflammatory actions, and immune modulation (55). This effect may result from the moderate oxidative stress signals (e.g., upregulation of azelaic acid) triggered by PMS supplementation, which activate cellular defense systems and subsequently regulate lipid metabolism pathways. In non-targeted metabolomics analyses, the sphingolipid signaling pathway and associated metabolites (DL-serine, sphingosine) were significantly upregulated in the S2 group compared to S1. This pathway acts as a central regulator of cellular stress, proliferation, and apoptosis, facilitating adaptive cellular remodeling and thereby enhancing PUFA and DHA synthesis (51).

Research has demonstrated that the composition and content of volatile flavor compounds are critical determinants of the sensory quality of meat products. These compounds predominantly arise from pathways such as lipid oxidation, the Maillard reaction, thiamine degradation, and microbial metabolism, collectively forming the characteristic aroma of meat and influencing consumer acceptance (56). Typically, aroma compounds in meat products encompass aldehydes, alcohols, ketones, esters, acids, among others (57). Esters are prevalent volatile flavor components in foods, with many ester compounds contributing desirable fruity aromas to meat products (58). Aldehydes, due to their high abundance and low detection threshold in meat products, generally play a pivotal role in shaping the overall volatile flavor profile of lamb, with most aldehydes emitting pleasant aromas at low concentrations (59).

In contrast, alcohols possess a relatively high detection threshold and thus have a minor impact on lamb flavor; however, elevated concentrations of ethanol can impart vanilla, woody, and fatty aromas to lamb. This study identified that methyl 3-methylpentanoate, a compound associated with fresh fruity aromas, exhibited the highest concentration in Groups S2 and S3. In contrast, methyl heptanoate, known for its fatty and fruity notes, reached its peak concentration in Group S2, demonstrating a significant increase of 62.89% compared to the control group. This observation is consistent with the findings of Bessa et al. (60), which elucidate the formation mechanism of branched-chain fatty acids in ruminants, suggesting that these acids are derived from the microbial metabolism of branched-chain amino acids, which serve as key precursors for the synthesis of branched-chain esters. Consequently, we propose that PMS supplementation may further influence the production of flavor compounds by modulating microbial metabolic pathways. Additionally, the concentrations of pentyl formate, decanal, (Z)-3-hexenal, 2-methylheptanoic acid, and 6-methyl-2-heptanone also increased with higher PMS dosages. Amaral et al. (61) demonstrated that maintaining appropriate mineral levels in muscle tissue effectively controls lipid oxidation by regulating antioxidant enzyme activity, thereby promoting the formation of desirable flavor compounds such as aldehydes, ketones, and alcohols. Based on these findings, we hypothesize that the addition of PMS may provide precursors for ester synthesis and regulate lipid oxidation, thereby enhancing the overall flavor quality of lamb. Subsequent screening using a VIP > 1 criterion identified 1-hydrazinopropan-2-ol, 2-octanol, and methyl 3-methylpentanoate as major contributors to lamb flavor. Notably, 1-hydrazinopropan-2-ol exhibited a high VIP value and showed a decreasing concentration with increasing PMS dosage. This compound is known to impart pungent odors (62) and is positively correlated with stress-related metabolites such as acetone aldehyde and sphingosine. This suggests that PMS addition may enhance sensory quality by inhibiting the formation of such undesirable flavor compounds. Concurrently, the VIP values for 2-octanol, which contributes a nutty aroma, and methyl 3-methylpentanoate, which contributes a fruity aroma, were also relatively high. Research has demonstrated that mineral salts play a crucial role in regulating lipid oxidation pathways by stabilizing cell membrane structures and reducing the production of hydroxyl radicals (63). The addition of PMS (presumably a specific mineral salt) has been shown to enhance the formation of desirable flavor compounds.

Supplementing the diets of Tibetan sheep with PMS was associated with improved meat quality and flavor by modulating key metabolites and synergistically influencing metabolic pathways. Specifically, the upregulation of metabolites such as N-acylsphingosine, (4E)-sphing-4-enine ceramide, capric acid, Cis-9-palmitoleic acid, and myristic acid was associated with the activation of adipokine signaling pathways and fatty acid biosynthesis pathways. This process promotes fat deposition while optimizing meat tenderness and juiciness, thereby enhancing the production of favorable flavor compounds. Simultaneously, the upregulation of metabolites such as DL-serine and palmitic acid was associated with the activation of the sphingosine signaling pathway, which further improves water-holding capacity and increases unsaturated fatty acid content, while also suppressing the formation of undesirable flavor compounds such as 2-propanol and 1-hydrazinopropan-2-ol. Ultimately, PMS was associated with significantly improved edible quality and flavor profile of Tibetan sheep meat through synergistic co-regulation.

The present study has several limitations. First, the experimental design cannot distinguish whether the observed effects of PMS arise from the individual actions of magnesium, potassium, and sulfate ions or from their synergistic interaction. Second, the metabolomics analysis was performed with a sample size of *n* = 6 per group, which may limit statistical power for detecting subtle metabolic changes. Therefore, the current findings are preliminary and warrant further validation with larger cohorts and factorial-designed experiments. Additionally, Tibetan sheep possess unique physiological adaptations to high-altitude environments, and caution should be exercised when extrapolating these findings to lowland sheep breeds without further comparative validation. Despite these limitations, this study provides the first comprehensive evidence that PMS supplementation is associated with the regulation of metabolic pathways involved in meat quality and flavor improvement in Tibetan sheep. Future studies should be designed to elucidate the individual and synergistic roles of each ion, validate the present findings with larger sample sizes, and compare the effects of PMS across different sheep breeds and production systems.

## 5 Conclusion

**Figure 10:**
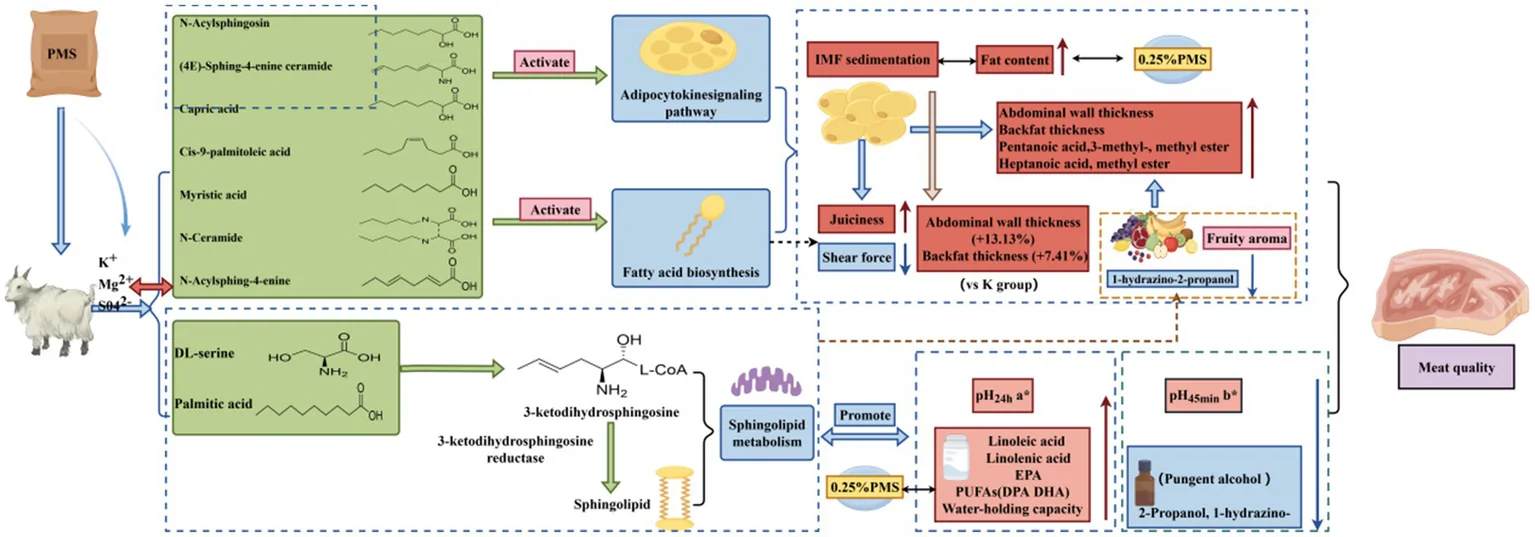
Potential mechanism diagram of meat quality, volatile metabolites, and non-volatile metabolites. The blue arrow represents the inhibition or decrease of phenotypic features during metabolic processes, while the red arrow represents the promotion or enhancement of phenotypic features during metabolic processes. Group K was fed with feed without PMS, Group S1 was fed with feed with 0.20% PMS, Group S2 was fed with feed with 0.25% PMS, and Group S3 was fed with feed with 0.30% PMS.

Dietary supplementation with magnesium potassium sulfate (PMS) was associated with significantly enhanced the overall quality of Tibetan sheep meat. Notably, PMS supplementation was associated with a marked increase in carcass fat deposition, with the 0.30% PMS group showing significantly greater abdominal wall and backfat thickness compared to the control group (*p* < 0.05). The 0.25% PMS group demonstrated significantly higher pH24h, a^*^, and intramuscular fat content. Furthermore, this group exhibited significantly elevated levels of linoleic acid, linolenic acid, EPA, and DHA relative to the control group (*p* < 0.05). Additionally, PMS supplementation was associated with improved flavor characteristics, which correlated with the production of desirable fruity compounds, such as methyl 3-methylpentanoate, while being associated with the suppression of undesirable flavor compounds like 2-propanol and 1-hydrazinopropan-2-ol. Non-targeted metabolomics revealed a systematic enhancement of the edible quality, nutritional value, and flavor properties of Tibetan sheep at the metabolic level, which was associated with the adipokine signaling pathway, fatty acid biosynthesis, and the sphingolipid signaling pathway. Compared between the 0.25 and 0.30% PMS groups, 0.25% PMS was more effective in improving meat quality, while 0.30% PMS was the most ideal for increasing carcass fat percentage. This study establishes a dependable methodological framework for the standardized production of high-quality Tibetan sheep meat (Figure 10).

## Data Availability

The raw data generated in this study have been submitted to the MetaboLights repository (https://www.ebi.ac.uk/metabolights/) under accession number MTBLS13926.
