# Correction: Effects of magnesium potassium sulfate on Tibetan sheep quality and its volatile and non-volatile metabolic substances

**DOI:** 10.3389/fnut.2026.2004912

**Published:** 2026-10-13

**Authors:** Yuan Wang, Lijuan Han, Shengzhen Hou, Linsheng Gui, Zhenzhen Yuan, Shengnan Sun, Zhiyou Wang, Baochun Yang, Chao Yang

**Affiliations:** College of Agriculture and Animal Husbandry, Qinghai University, Xining, China

**Keywords:** meat quality, potassium magnesium sulfate, Tibetan sheep, untargeted metabolomics, volatile flavor compounds

There was a mistake in Table 2 as published. The mean values and SD values of three body measurement indicators (Body height, Bust, Slant length) for groups S1, S2 and S3 were written incorrectly. The corresponding *p*-values for these indicators remain unchanged. This is a data-entry typographical error and does not affect the main results and conclusions of the present study.

The corrected Table 2 appears below.

**Table 2:** Effect of different levels of PMS on carcass quality of Tibetan sheep.

Project	Group				*p*-value
	K	S1	S2	S3	
Body height/cm	65.32 ± 2.50	68.47 ± 2.51	72.58 ± 7.39	67.38 ± 2.57	0.086
Bust/cm	83.93 ± 2.77	81.42 ± 3.90	83.92 ± 3.32	80.83 ± 3.00	0.320
Slant length/cm	72.92 ± 2.76	74.92 ± 3.55	75.33 ± 4.78	76.43 ± 3.23	0.505
Live weight/kg	34.68 ± 0.85^b^	35.37 ± 0.66^b^	35.43 ± 0.48^b^	36.34 ± 0.58^a^	0.007
Carcass weight/kg	16.52 ± 0.52	15.77 ± 0.84	16.03 ± 0.51	16.78 ± 0.40	0.055
Slaughter rate/%	47.62 ± 0.62	44.56 ± 1.74	45.25 ± 1.13	46.10 ± 0.65	0.060
Eye muscle area/cm^2^	21.33 ± 3.20	16.50 ± 2.99	21.00 ± 2.24	22.50 ± 2.75	0.080
Rib thickness/mm	25.33 ± 0.66	22.25 ± 1.35	23.78 ± 1.89	26.11 ± 2.12	0.070
Abdominal wall thickness/mm	20.80 ± 1.30^bc^	18.78 ± 1.51^c^	22.09 ± 1.61^ab^	23.53 ± 1.07^a^	< 0.01
Backfat thickness/mm	20.23 ± 1.14^b^	14.78 ± 1.68^c^	18.33 ± 1.24^b^	21.73 ± 1.99^a^	< 0.01
ADG/(kg/d)	0.21 ± 0.02	0.23 ± 0.01	0.22 ± 0.01	0.23 ± 0.01	0.066
FCR	6.38 ± 0.49^a^	5.68 ± 0.30^b^	6.04 ± 0.38^ab^	5.99 ± 0.36^ab^	0.037
ADFI/(kg/d)	1.29 ± 0.33^c^	1.35 ± 0.31^b^	1.39 ± 0.33^a^	1.35 ± 0.30^b^	< 0.01

ADG refers to average daily gain, FCR refers to feed conversion ratio and ADFI refers to the average daily feed intake. Different letters indicate significant differences between groups after dietary supplementation with different levels of PMS (*p* < 0.05). Group K was fed a basal diet without PMS, Group S1 was fed a diet supplemented with 0.20% PMS, Group S2 was fed a diet supplemented with 0.25% PMS, and Group S3 was fed a diet supplemented with 0.30% PMS.

The original version of this article has been updated.

