# Supplementary material for: Effects of magnesium potassium sulfate on Tibetan sheep quality and its volatile and non-volatile metabolic substances
Source: Front Nutr. 2026 May 11;13:1821555. doi: 10.3389/fnut.2026.1821555 (PMC13199345; doi:10.3389/fnut.2026.1821555)
Supplement: Supplementary file 1 [file Supplementary_file_1.docx]

**1 Supplementary Figures and Tables**

**1.1 Supplementary Tables**

**Supplementary Table S1.** Sensory evaluation table of Tibetan sheep meat

|  | Sensory score | | |
| --- | --- | --- | --- |
|  | 15-20 points | 10-14 points | 0-9 points |
| Exterior | Muscle fibers are clearly defined and dense, with abundant fat | Muscle fibers are well-defined with minimal fat content | The muscle fibers are very loose and free of fat |
| Organizational Status | Muscle fibers are dense, resilient, and highly elastic | Muscle fibers are lax, with low toughness and average elasticity | Muscle fibers soften, losing elasticity and resilience |
| Flavor | It has a rich, savory flavor | The flavor is average | Completely lacking in savory flavor |
| Juicy feeling | Juicy and plump, bursting with juice upon the first bite | Juicy, with a juicy sensation after chewing 4 to 6 times | Very little juice, even after chewing over ten times, there is still no juicy sensation |
| Overall Evaluation | Overall, it feels great | Overall, it's just okay | Overall, it feels subpar |

**1.2 Supplementary Figures**


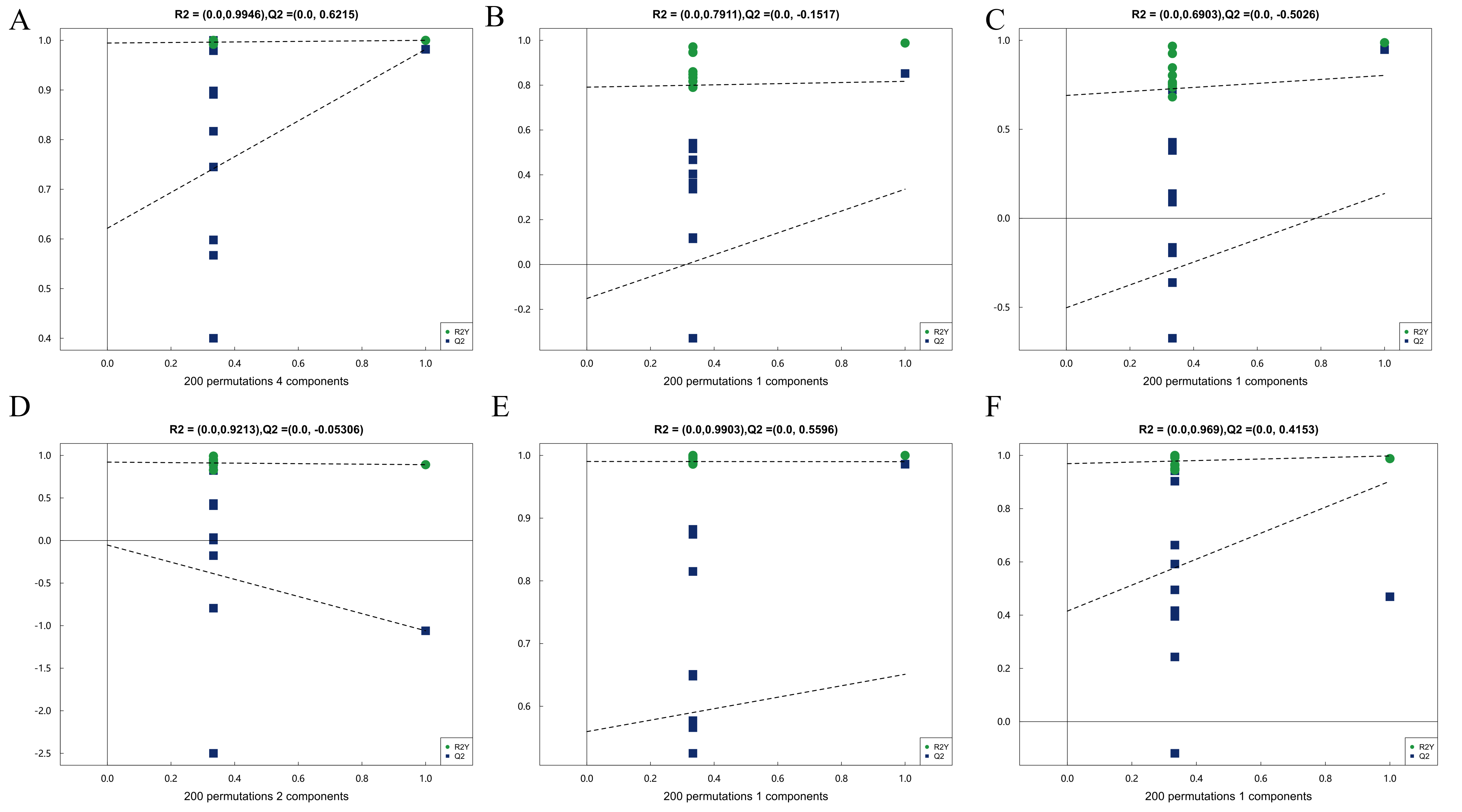


**Supplementary Figure S1.** Substitution test diagrams for volatile compounds in Tibetan sheep with different PMS treatments (A: K vs. S1; B: K vs. S2; C: K vs. S3; D: S1 vs. S2; E: S1 vs. S3; F: S2 vs. S3) (A–F) Permutation test plots of the PLS-DA models for different comparison groups. The R² and Q² values of the original model are represented by green dots, while the R² and Q² values after 200 random permutations are represented by blue dots. The dashed lines represent the regression lines of R² and Q² after permutation. R² indicates the explanatory power of the model, and Q² indicates the predictive power of the model.


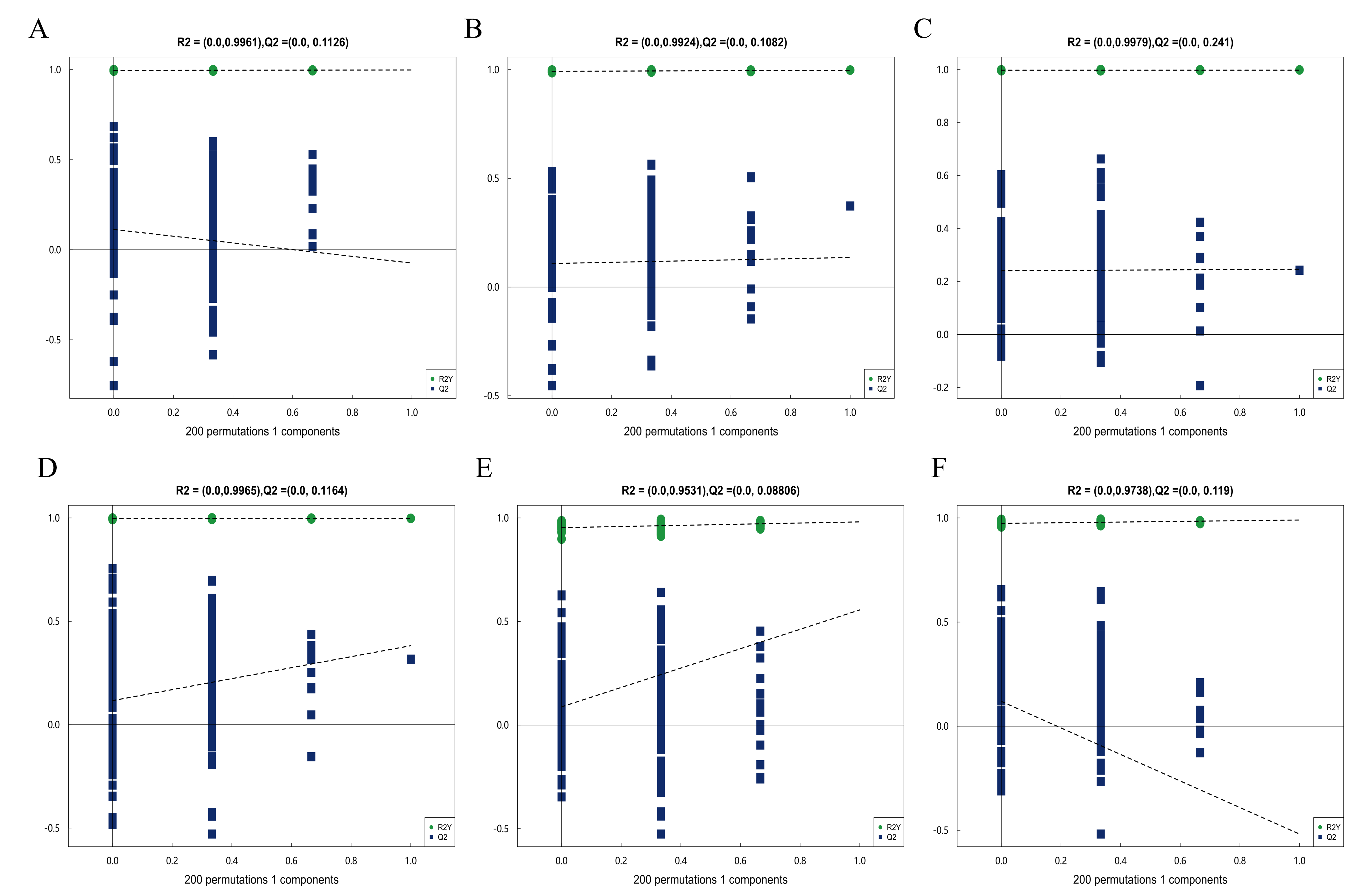


**Supplementary Figure S2.** Substitution Test Diagram of Non-Targeted Metabolites in Tibetan sheep Meat with Different PMSs (A: K vs. S1; B: K vs. S2; C: K vs. S3; D: S1 vs. S2; E: S1 vs. S3; F: S2 vs. S3) (A–F) Permutation test plots of the PLS-DA models for different comparison groups. The R² and Q² values of the original model are represented by green dots, while the R² and Q² values after 200 random permutations are represented by blue dots. The dashed lines represent the regression lines of R² and Q² after permutation. R² indicates the explanatory power of the model, and Q² indicates the predictive power of the model.


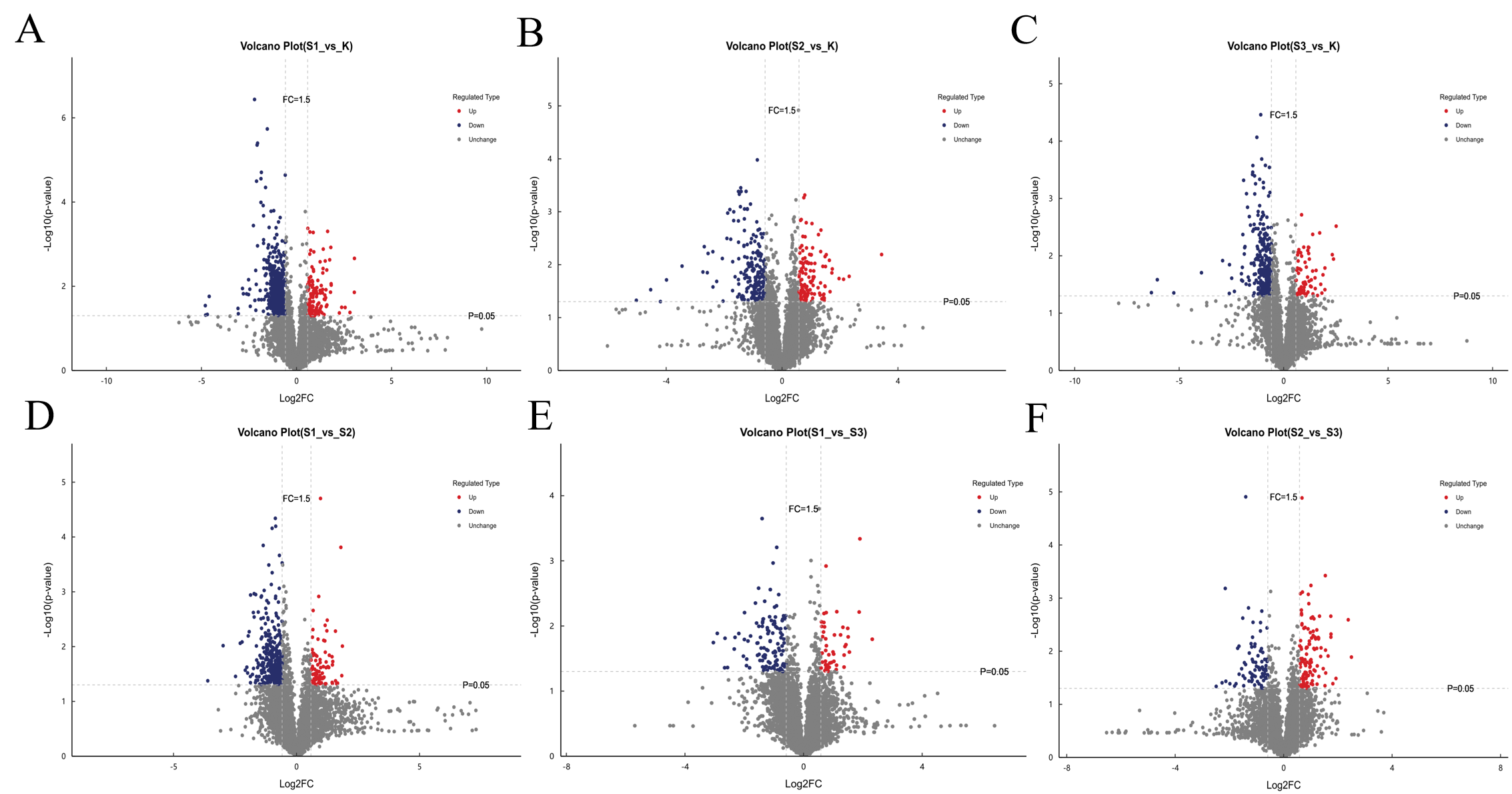


**Supplementary Figure S3.** Volcanic diagrams in positive ion mode between four pairs (A: K and S1, B: K and S2, C: K and S3; D: S1 and S2; E: S1 and S3; F: S2 and S3) (A–F) Volcano plots showing the changes in metabolite abundance between the indicated groups. Red dots represent significantly upregulated metabolites (*P* < 0.05), blue dots represent significantly downregulated metabolites (*P* < 0.05), and gray dots represent non-significant metabolites.


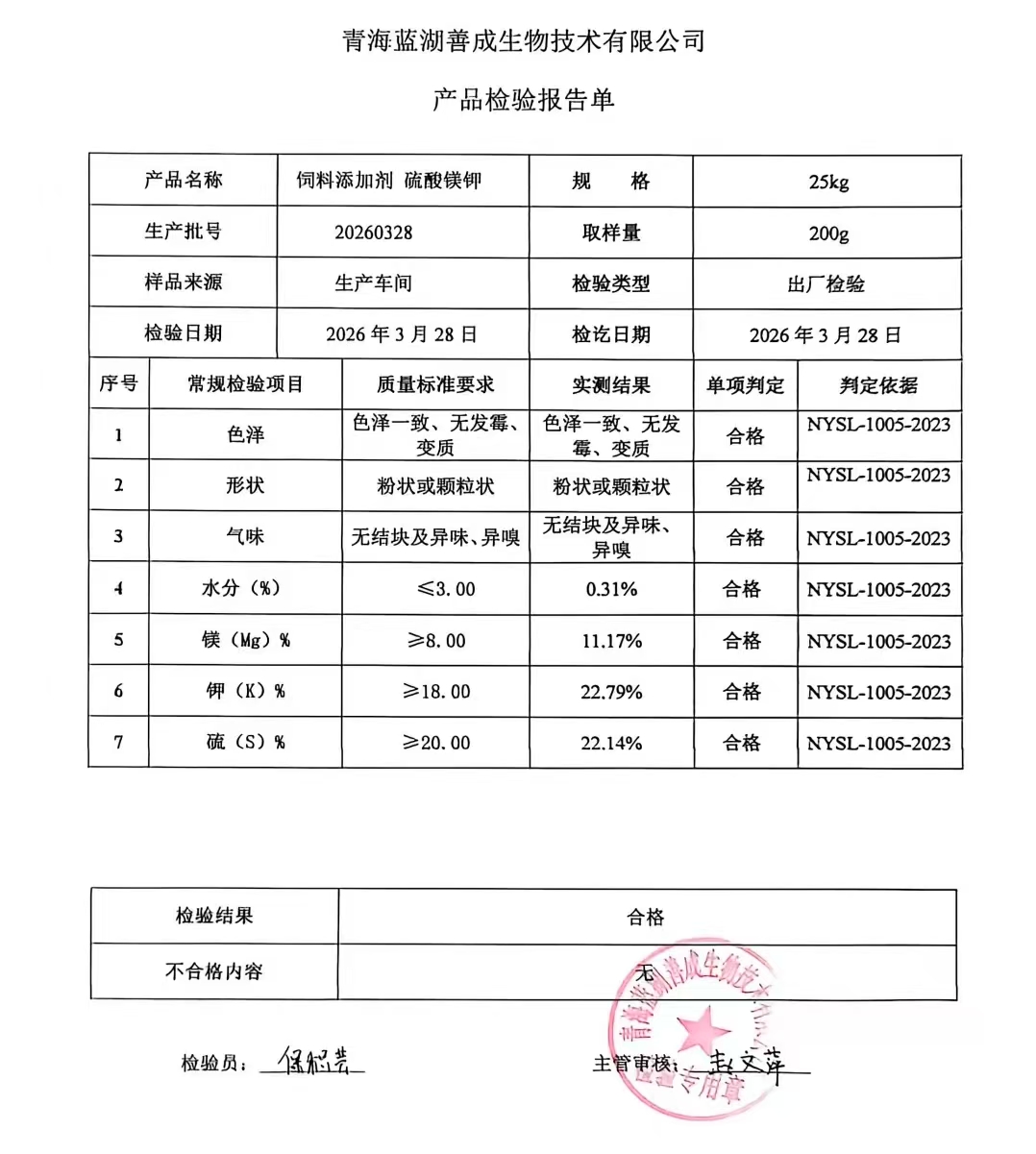


**Supplementary Figure S4.** PMS analysis proof materials
